# From C─H Bond Insertion to Hydrogen Atom Transfer: Tuning the Reaction Mechanisms of Methane Activation by the Oxidation of Ta_2_^+^

**DOI:** 10.1002/chem.202500545

**Published:** 2025-05-06

**Authors:** Flora Siegele, Jan F. Eckhard, Tsugunosuke Masubuchi, George Goddard, Detlef Schooss, Dmitry I. Sharapa, Felix Studt, Martin Tschurl, Ueli Heiz

**Affiliations:** ^1^ Lehrstuhl für Physikalische Chemie I Technische Universität München, School of Natural Sciences Lichtenbergstraße 4 85748 Garching Germany; ^2^ Institute of Nanotechnology Karlsruhe Institute of Technology (KIT) Kaiserstraße 12 76131 Karlsruhe Germany; ^3^ Institut für Katalyseforschung und –technologie (IKFT) Hermann‐von‐Helmholtz‐Platz 1 76344 Eggenstein‐Leopoldshafen Germany; ^4^ Institute for Chemical Technology and Polymer Chemistry (ITCP) Karlsruhe Institute of Technology (KIT) Engesserstr. 20 76131 Karlsruhe Germany

**Keywords:** DFT calculations, ion‐molecule reactions, methane activation, oxidation, tantalum

## Abstract

The activation of methane under mild conditions is a challenging but rewarding goal; the underlying key parameters, however, remain elusive. In this study on isolated tantalum Ta_2_^+^ compounds exposed to methane in a ring‐electrode ion trap, strong changes in the reactivity are observed depending on the compound's degree of oxidation. While the general reaction behavior is presented for species ranging from Ta_2_^+^ to Ta_2_O_6_^+^ based on experimental kinetic studies, we focus in more detail on the dehydrogenation reactions occurring on Ta_2_O_2_^+^ and the hydrogen atom transfer (HAT) on Ta_2_O_5_^+^, for which density functional theory calculations were performed. In the first part, we elucidate the role of Ta–C–Ta bridging motifs in product structures as driving forces for the dehydrogenation of methane on Ta_2_O_2_^+^; in the second part, we investigate the origins of the HAT – a hitherto unknown reaction scheme for binary tantalum oxides. For the latter, we show that the reactivity originates from the spin density on oxygen atoms, which is a typical characteristic of the reaction on other metal oxides. This reflects a change in the reactivity from oxidized metallic systems to metal oxides and demonstrates that chemical modifications of tantalum compounds can achieve different methane activation schemes.

## Graphical abstract

In a combined experimental and theoretical study, it is observed that the continued oxidation from Ta_2_^+^ to Ta_2_O_x_^+^ (with *x* = 1 – 6) changes the reactivity with methane from a typical metallic to a typical oxidic behavior. While the bridging motifs in Ta_2_O_2_^+^ enable the dehydrogenation of CH_4_, the spin density on oxygen atoms in Ta_2_O_5_^+^ facilitates a HAT reaction.

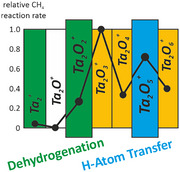

## 1 Introduction

With the concern of climate change being more topical now than ever holding back on the emission of greenhouse gases like methane becomes more and more important. Besides the prevention of newly emitted pollutants, another approach is to decrease the already existing environmental pollution by recycling these harmful chemicals into more valuable compounds. Because of methane's stability due to its intrinsic molecular properties like the strong sp^3^‐hybridization of the C─H bond, the small pK_a_‐value, and its small polarizability, the activation of the molecule is very challenging under mild conditions.^[^ ^1^ ^]^ The two most common catalytic conversion processes of methane are its full combustion and steam reforming of methane (SRM). Both reactions lead to the formation of large amounts of CO_2_, which is an unwanted greenhouse gas itself.^[^ ^2^ ^]^ Different from the full combustion, the partial oxidation in SRM performed for yielding H_2_ does not necessarily cause the unwanted by‐product CO_2_.^[^ ^3^, ^4^ ^]^ However, for changing this pathway away from the production of CO_2_, a comprehensive understanding of the interaction of the catalyst with methane is crucial to enable a tailored catalyst design. This knowledge is even more important when catalysts are developed to make one of the other methane reformation pathways technologically feasible.

In heterogeneous catalysis, tantalum compounds have been shown to enable the nonoxidative coupling of methane at considerably low temperatures with the conversion readily approaching the thermodynamic limit.^[^ ^5^ ^]^ As this reaction yields ethane and hydrogen, it represents a very interesting pathway for a chemical transformation of methane into substances of further use. While the authors aimed at elucidating key aspects of the catalyst and the reaction mechanism, many open questions remain. Answers may be gained from studies of simpler systems, which can be understood more in‐depth. Indeed, in ion‐molecule reactions of trapped clusters in the gas phase as the most simplified model system, we observed a reaction on Ta_8_O_2_^+^ clusters,^[^ ^6^ ^]^ which similarly occurs in heterogeneous catalysis on the previously mentioned tantalum‐based catalyst.^[^ ^5^ ^]^ In general, different experiments performed with isolated cationic tantalum compounds (either under multi‐collision, i.e., isothermal, or single‐collision conditions) have clearly demonstrated the potential of tantalum species in enabling the reaction with methane via C─H bond activations.^[^ ^7^ ^]^ Experimental, theoretical, and combined studies revealed that already the atomic cation (i.e., Ta^+^) facilitates the dehydrogenation reaction of methane.^[^ ^8^, ^9^, ^10^, ^11^, ^12^, ^13^ ^]^ The same reaction still proceeded on Ta_2_^+^, Ta_3_^+^, and Ta_4_^+^ but vanished on larger cluster cations (i.e., Ta_n_^+^, *n* = 5–10).^[^ ^14^ ^]^ The oxidation of the compounds resulted in a change in their reactivity. For cationic compounds with one Ta‐atom, TaO_3_^+^ allowed the reaction with methane to occur via three different channels.^[^ ^15^ ^]^ In contrast, TaO_2_^+^ was rather unreactive and the interaction with methane proceeded without releasing any neutral species, while theory suggested the activation of the C─H bond.^[^ ^16^ ^]^ In one of our studies, adding a single oxygen atom to Ta_4_^+^ and the formerly unreactive Ta_5_^+^ greatly improved the reactivity in methane dehydrogenation.^[^ ^17^ ^]^ Theory suggested that the oxygen atom functions as a ligand on these clusters, which varies the clusters’ electronic properties but does not actively participate in the reaction by bond formations with the hydrocarbon. While the origin of the reactivity remained elusive, a recent study by Li et al. assigned it to the presence of localized σ‐bonding orbitals, which was concluded from the reaction behavior of cationic Ta‐clusters of different sizes and degrees of oxidation with methane.^[^ ^18^ ^]^ In that study, the authors also examined the reaction of Ta_2_^+^ oxides, but only up to Ta_2_O_4_^+^ and primarily for the interaction with the first CH_4_ molecule. In all those studies, however, the hydrocarbon was observed to approach a tantalum atom with the carbon atom in the first activation step. In case of an activation, a C─H bond cleavage resulted and the reaction may thus be seen as an insertion of the metal (i.e., Ta) into the C─H bond. Different from other metal oxides,^[^ ^19^, ^20^, ^21^, ^22^, ^23^, ^24^ ^]^ other reaction schemes, such as a proton‐coupled electron transfer (PCET) or a hydrogen atom transfer (HAT) have so far not been described on binary cationic tantalum oxide compounds in the gas phase.

In the following, we report the reaction of methane with cationic Ta_2_ compounds with a different number of oxygen atoms. Thereby, we focus on the reaction of Ta_2_O_5_^+^ and the carbonaceous products formed in the reaction of Ta_2_O_2_^+^, because both topics have not been reported in the literature yet, despite their relevance for a comprehensive understanding of pathways and mechanisms in the activation of methane.

## 2 Methods

### 2.1 Experimental Section

All experiments are performed in a high‐vacuum chamber with base pressures ranging from 5 × 10^−4^ Pa for the cluster generation to < 2 × 10^−7^ Pa in the reaction region, and 2 × 10^−6^ Pa in the analysis part of the apparatus. Tantalum clusters are generated in a laser vaporization cluster source, which is an adaptation of the original design by Smalley et al.^[^ ^25^ ^]^ by Heiz et al.^[^ ^26^ ^]^ An additional modification allows for the controlled chemical transformation of the clusters (here, their oxidation) in a second reaction zone (also called “waiting room”) inside the cluster source.^[^ ^27^ ^]^ For the ablation, a 100 Hz laser (532 nm, Innolas Spitlight DPSS) is used. In the first waiting room, helium (He 5.0, Westfalen) is added as a buffer and carrier gas acting as a heat bath for thermalization and enabling the formation of clusters. When the clusters enter the second waiting room, they can react with oxygen (O_2_ 5.5, Air Liquide), which may be added by a gas pulse from a solenoid valve. This way, the formation of oxidized tantalum clusters is enabled.^[^ ^27^ ^]^ When leaving the waiting room, the clusters undergo expansion into the vacuum. Einzel lenses guide the clusters through the setup. A quadrupole bender is used to achieve the separation of charged from neutral species. For size selection of the single‐charged compounds formed in the cluster source, the cluster beam passes a quadrupole mass filter (model 5221, Extrel) before it enters a home‐built, temperature‐controlled ring electrode ion trap (REIT)^[^ ^28^ ^]^ adapted from the designs of Gerlich^[^ ^29^ ^]^ and Goebbert et al.^[^ ^30^ ^]^ The REIT can be cooled down using a closed‐circuit helium cryostat (RW2, Leybold), which is connected to the REIT via a cold head. All experiments in this work, however have been performed at room temperature (RT) Via a mass flow controller (500 sccm range, MKS), helium as a buffer gas (He 6.0, Westfalen), and a defined fraction of the reactant methane (CH_4_ 5.5, Rießner Gase and CD_4_ 99.9% isotope enrichment, Eurisotop; respectively) being premixed in a separate mixing chamber can be introduced into the REIT. In the experiments presented here, the reactant gas was diluted with He to a concentration of 0.05%. The pressure in the trap is thereby monitored by a capacitance manometer (Baratron model 722B, MKS) and set to 0.77 Pa using the mass flow controller. Due to the pressure within the REIT, the clusters are thermalized quickly (within one millisecond^[^ ^31^ ^]^) by colliding approximately 100 times per millisecond with the buffer gas. The use of the buffer gas He also enables performing the reactions under multicollision conditions, which allows for overcoming barriers above the energy of the reactants and the stabilization of intermediates.^[^ ^32^ ^]^ The trap enables the storage of the clusters from a few milliseconds up to the range of several seconds, which corresponds to the reaction time. After a given storage time, the clusters are ejected into a home‐built reflectron time‐of‐flight mass spectrometer (re‐ToF MS), which allows the determination of the mass‐to‐charge ratio (m/z) of all charged species as well as their abundance.^[^ ^28^ ^]^ The formation of neutral species, for example the evolution of H_2_ can instead only be deduced from the detected charged products, for example from the presence of CH_2_ fragments on the tantalum clusters. A kinetic study of the reactions is conducted by varying the storage time in the REIT systematically and evaluating the data by plotting the abundance of each reaction product against the respective reaction time. This forms the basis for kinetic simulations, whereby different chemically feasible reaction models are compared. The simplest model yielding a good correspondence is assumed to be correct to describe the kinetics of the process. A more detailed description of the experimental procedures can be found in our previous works, such as in Eckhard et al.^[^ ^14^ ^]^

### 2.2 Computational Details

All calculations using density functional theory (DFT) were carried out with Orca 5.0.3 on the LRZ Linux‐Cluster CoolMUC‐2. The TPSS functional,^[^ ^33^ ^]^ def2TZVP^[^ ^34^ ^]^ basis set, and Grimme's dispersion correction (DFT‐D3)^[^ ^35^ ^]^ were used, similar as in other studies.^[^ ^36^ ^]^ All calculations were performed with the resolution of identity approximation (RIJ).^[^ ^37^ ^]^ In order to characterize calculated structures as ground or transition states (TS), analytical IR spectra of all structures are calculated whereby only positive vibrational frequencies correspond to a minimum structure and one negative frequency hints toward a TS structure. All reported energies are corrected by zero‐point energy (zpe) as well as thermal and entropy parts to compare final Gibbs free energies at a temperature of 298.15 K and a pressure of 1 atm (1.013 · 10^5^ Pa). Potential energy calculations of all structures were carried out for the lowest three multiplicities to check for the present multiplicity and multiplicity changes during the reaction. The reaction path was determined using relaxed surface scans, whereby the reaction coordinates were altered in small increments. From following the reaction path, local minima and barrier configurations were determined, whereby the structure with the maximum energy on the potential energy surface was selected as a possible TS candidate and optimized concerning the negative vibration frequency.

## 3 Results and Discussion

**Figure 1:**
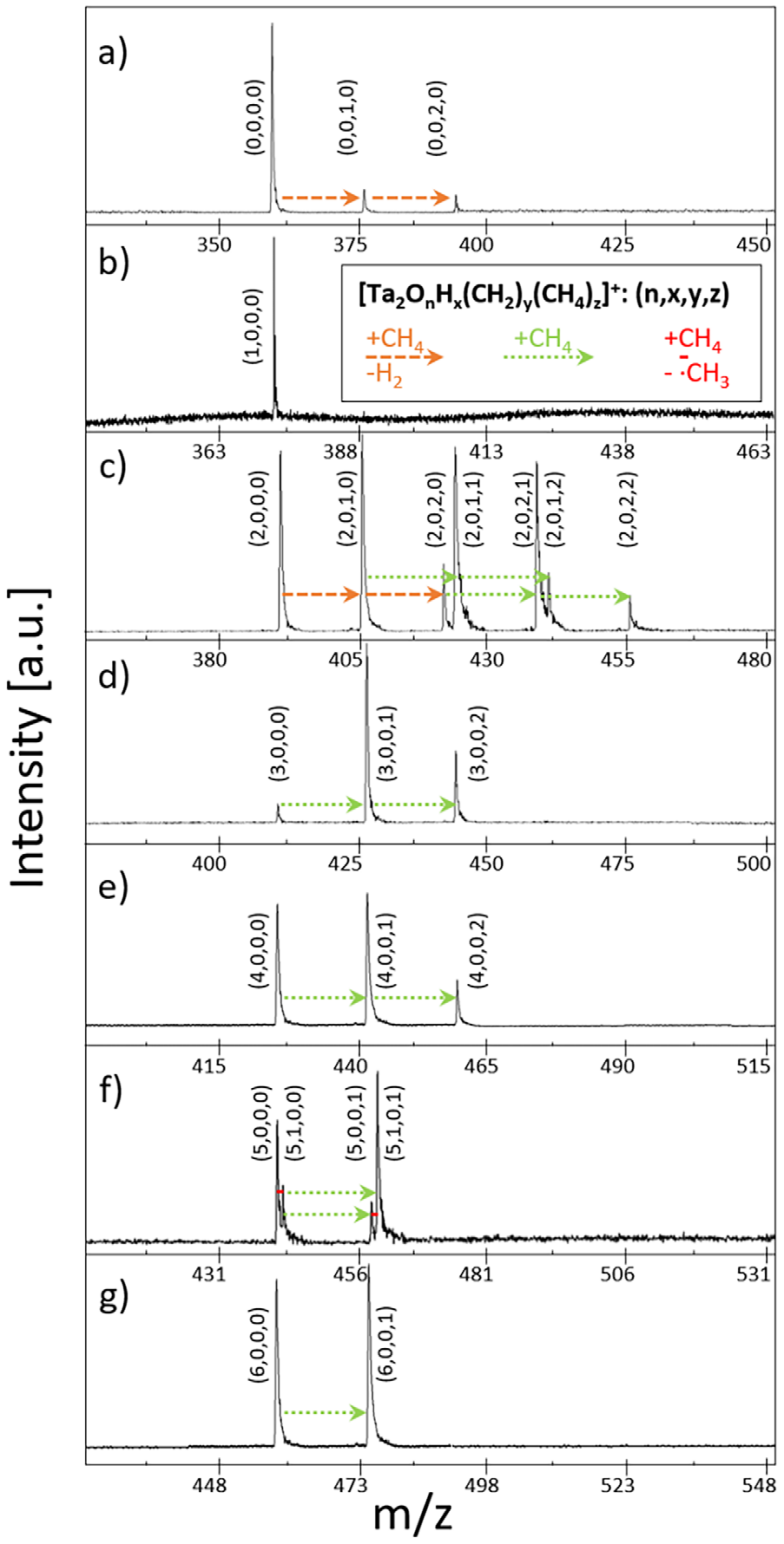
Mass spectra of Ta_2_O_n_^+^ with an increasing number of oxygen atoms (*n*) from a (*n* = 0) to g (*n* = 6) in the reaction with CH_4_. Dashed orange arrows indicate the dehydrogenation of methane, resulting in an addition of CH_2_ (m/z = 14) to the cluster after the release of H_2_. Dotted green arrows show the adsorption of CH_4_ (m/z = 16) and solid red lines denote the addition of a single hydrogen atom (m/z = 1) by releasing a methyl radical. To show all detected species occurring during the course of the reactions, the mass spectra represent an average over several storage times. It is found that the majority of oxides only react via the adsorption of entire CH_4_ molecules. The exceptions are Ta_2_^+^ and Ta_2_O_2_^+^, which enable the dehydrogenation of methane in two consecutive reactions and Ta_2_O_5_^+^ on which a HAT from CH_4_ to the cluster occurs.

As expected from literature, the reaction of cationic Ta_2_ compounds with methane strongly depends on the number of oxygen atoms bound to the metal atoms (see Figure 1). From the mass spectra, it becomes apparent that only Ta_2_^+^, Ta_2_O_2_^+^, and Ta_2_O_5_^+^ definitively lead to an activation of the C─H bond and its cleavage, while Ta_2_O^+^ proves to be inactive and Ta_2_O_3_^+^, Ta_2_O_4_^+^, and Ta_2_O_6_^+^ just add CH_4_. These observations are in very good agreement with a recent study by Li et al.^[^ ^18^ ^]^ In both works, Ta_2_^+^, Ta_2_O_2_^+^, Ta_2_O_3_^+^, and Ta_2_O_4_^+^ show the same reactivity and follow the same trends (see the rate coefficients of the reaction in Table S1 in the Supporting Information), namely dehydrogenation reactions on the first two and molecular methane adsorption on the latter two species. Only for Ta_2_O^+^, the results deviate, as no activity is observed in our experiments, while methane is dehydrogenated in the experiments of Li et al.^[^ ^18^ ^]^ However, the reaction rate reported in that study is more than two orders of magnitude lower than the one of the bare metal clusters, which makes the species effectively unreactive in comparison between Ta_2_^+^ and Ta_2_O_2_^+^. Due to the inferior reactivity of Ta_2_O^+^, Ta_2_O_3_^+^, Ta_2_O_4_^+^, and Ta_2_O_6_^+^ and the available data in the literature for Ta_2_^+^,^[^ ^14^, ^18^ ^]^ we omit these species from more detailed investigations. Instead, we focus on the underlying reaction mechanisms and products of the two methane dehydrogenation reactions on Ta_2_O_2_^+^ and the so far unexplored reaction of methane with Ta_2_O_5_^+^ in the following.

### 3.1 Ta_2_O_2_^+^+CH_4_ and the Structure of Reaction Products

**Figure 2:**
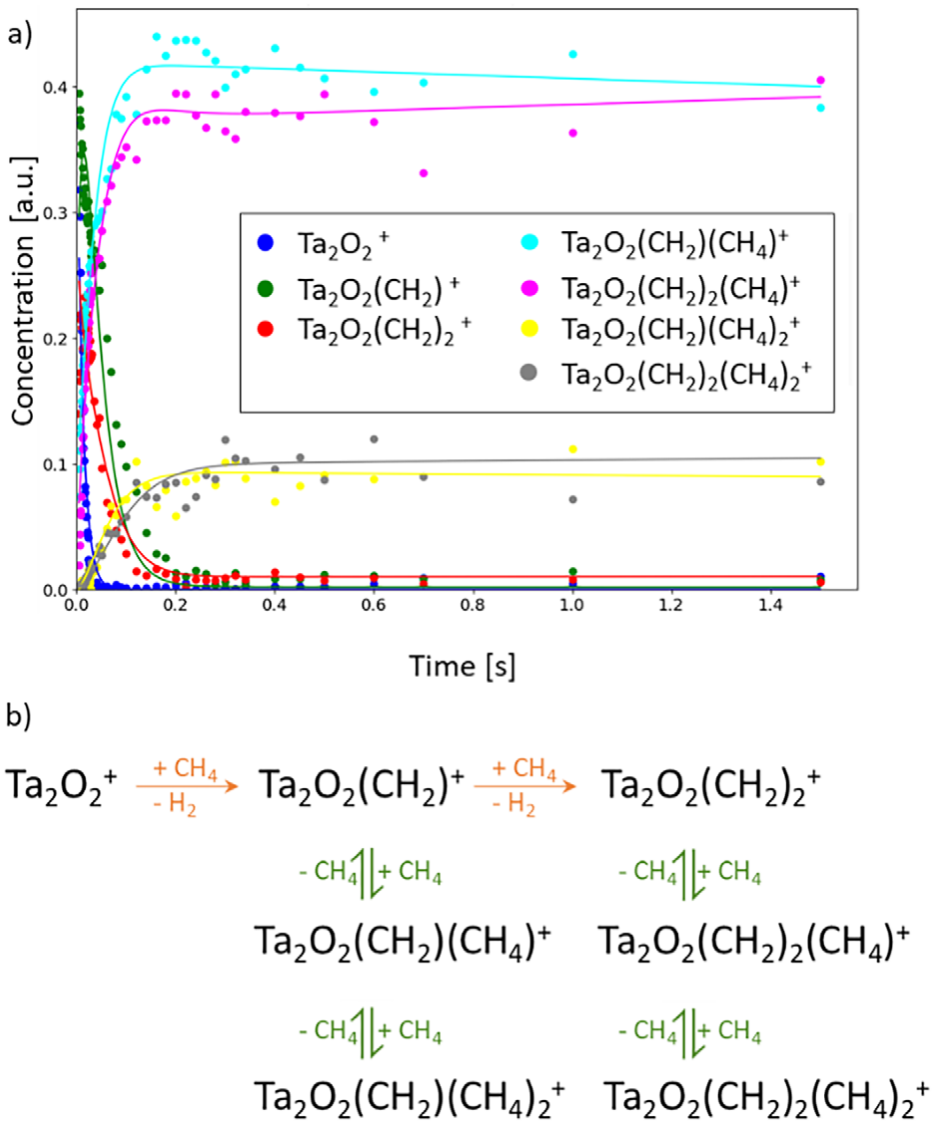
a) The fits of the kinetic simulations (lines) are shown in the respective color of the measured data points (dots) for each measured storage time. The fit is obtained with the reaction model shown in b).

The mass spectrum (Figure 1) shows signals for Ta_2_O_2_^+^ (m/z = 394), Ta_2_O_2_(CH_2_)^+^ (m/z = 408), Ta_2_O_2_(CH_2_)_2_^+^ (m/z = 422), Ta_2_O_2_(CH_2_) (CH_4_)^+^ (m/z = 424), Ta_2_O_2_(CH_2_) (CH_4_)_2_^+^ (m/z = 440), and Ta_2_O_2_(CH_2_)_2_(CH_4_)_2_^+^ (m/z = 454). No indication of the presence of Ta_2_O_2_(CH_4_)^+^ (m/z = 410) is found, which suggests that the first dehydrogenation takes place too fast for the intermediate to be detected. As the conversion of methane is very low due to the low density of Ta_2_O_2_^+^ in the ion trap in comparison to the reactant methane, the probability of a reaction with neutral products is negligible after their desorption from the charged species. Consequently, dehydrogenation reactions can thus be seen as formally irreversible due to the low density of H_2_ in the REIT during the reaction time. This is also considered in the kinetic fits (Figure 2a), where dehydrogenation reactions are assumed to be irreversible, while all molecular adsorption steps are fitted as reversible reaction steps. The model (Figure 2b) shows that two methane molecules can be dehydrogenated in two consecutive reaction steps. In addition, two reversible molecular adsorption steps can occur after each dehydrogenation reaction. The fits also suggest that the detected species of intact methane adsorption are not intermediates of the second dehydrogenation reaction. Again, the reaction occurs on a timescale too fast for the intermediates to be visible in the mass spectra. After the adsorption of an intact CH_4_ molecule (e.g., on a different site of the compound, as shown in Figure S13) the release of H_2_ is not possible anymore. The addition of this CH_4_ may prevent a restructuring of the species, sterically hinder the reaction with another CH_4_ molecule or quench the dehydrogenation before H_2_ can be evolved. The fits evidence such a reaction sequence, as the inclusion of additional pathways in the reaction model causes the rate coefficients of these reactions to approach zero, while simultaneously no improvement in the quality of the fits is obtained (compare Figure S2). Consequently, Ta_2_O_2_(CH_2_)_2_(CH_4_)^+^ and Ta_2_O_2_(CH_2_)_2_(CH_4_)_2_^+^ are only obtained by consecutive adsorption reactions on Ta_2_O_2_(CH_2_)_2_^+^ and not through a pathway via Ta_2_O_2_(CH_2_) (CH_4_)^+^ and Ta_2_O_2_(CH_2_) (CH_4_)_2_^+^. After an interaction with a maximum of four methane molecules, the reaction stops. Eventually, about equal amounts of products from one and two dehydrogenation reactions are yielded. In addition, the majority of species (about 80%) have adsorbed one intact CH_4_ molecule, while only about 20% carry two intact methane molecules.

The assignment of the species is further confirmed by experiments with isotopically labeled CD_4_ (data shown in the Supporting Information Figure S5 together with all values in Table S1). The comparison of the (bimolecular) rate coefficients of the dehydrogenation reactions with both isotopologues enables the calculation of kinetic isotope effects (KIE). The results are shown in Table 1 together with the rate coefficients determined by Li et al., demonstrating the good agreement of the results from both studies.^[^ ^18^ ^]^ It is found that the second dehydrogenation is considerably slower than the first one. For both reactions, similar and rather high KIE values of 5–6 are obtained, which is also observed in reactions of larger cationic Ta compounds, but differs from the reaction of the atomic Ta cation.^[^ ^7^, ^14^ ^]^ Such high values usually suggest an important contribution of H‐bond cleavages in the rate‐determining step,^[^ ^38^, ^39^ ^]^ while in these cases new bonds to the tantalum compound are formed.

**Table 1:** Bimolecular rate coefficients, given in 10^−11^ cm^3^s^−1^, and KIEs of the respective dehydrogenation reaction with the respective reactant (i.e., CH_4_ and CD_4_). Literature values are taken from^[^ ^18^ ^]^

Reaction	$k_{+,CH4}^{\left(2\right)}$	$k_{+,CH4,Lit}^{\left(2\right)}$	$k_{+,CD4}^{\left(2\right)}$	KIE
Ta_2_O_2_^+^ → Ta_2_O_2_(CH_2_)^+^	24.5 ± 5.2	22	3.71 ± 0.75	5.9 ± 1.7
Ta_2_O_2_(CH_2_)^+^ → Ta_2_O_2_(CH_2_)_2_^+^	2.62 ± 0.57	‐	0.465 ± 0.097	5.1 ± 2.2

**Figure 3:**
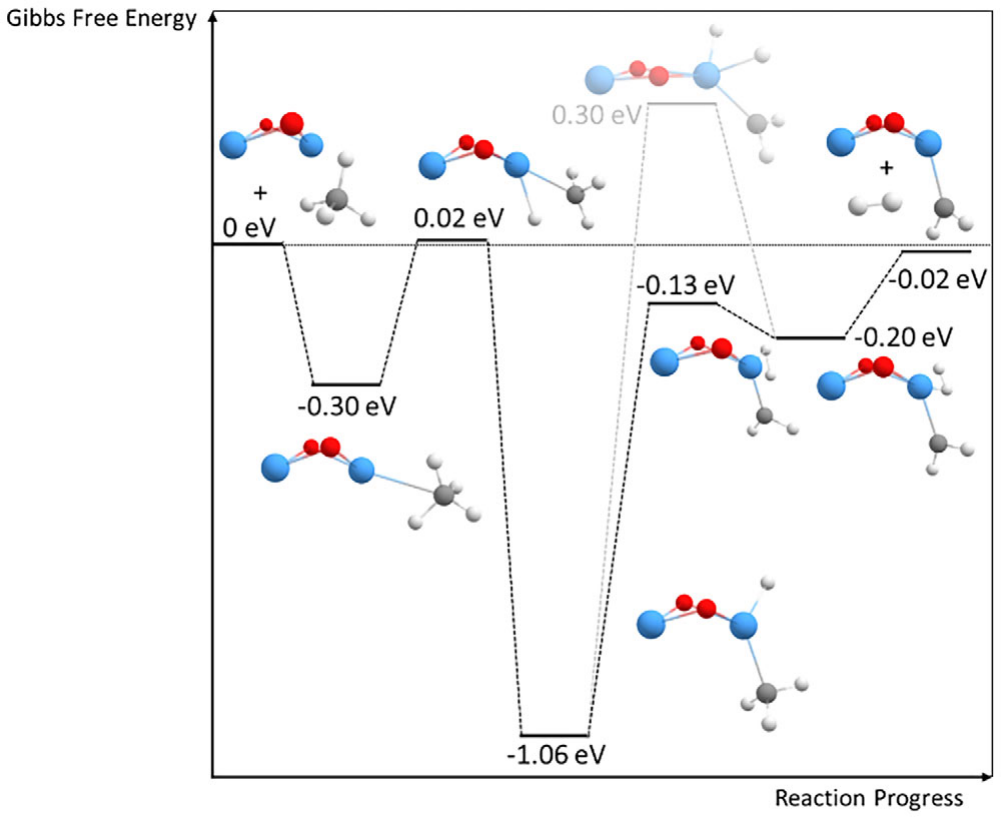
Energy scheme of the first dehydrogenation of CH_4_ on Ta_2_O_2_^+^ clusters (Gibbs free energies calculated for *T* = 298.15 K and p = 1 atm). All energies are determined using DFT energy optimizations. The energy of the bare Ta_2_O_2_^+^ with a free methane molecule is normalized to 0 eV. Grayed‐out is a different second transition state of higher energy that includes a further metal insertion to break the second C─H bond. Tantalum atoms are shown in blue, oxygen atoms in red, carbon atoms in gray, and hydrogen atoms in white.

To investigate the reaction mechanism and resulting product structures in more detail, DFT calculations of both dehydrogenation reactions were performed. The obtained structure for Ta_2_O_2_^+^ (Figure 3) is in very good agreement with the one published by Li et al.^[^ ^18^ ^]^ For this and all following structures in this section, the spin doublet state proves to be the most stable multiplicity (see Supporting Information Table S2 and S3). The first step in the dehydrogenation of methane involves the adsorption of the methane molecule. The carbon atom of the methane group thereby interacts with one of the tantalum atoms, independent of the direction from which methane approaches.

Due to the symmetry of the metal oxide cluster both tantalum atoms are equally likely to interact with the hydrocarbon. The adsorption of methane leads to an energy gain of approximately 0.30 eV. Inserting a tantalum atom into one of the C─H bonds of the methane molecule leads to the elongation of this bond and eventually, its cleavage. During this process, only a small energy barrier calculated to be around 0.02 eV relative to the initial energy of the bare Ta_2_O_2_^+^ and a free methane molecule must be overcome. The as‐formed intermediate represents the global energetic minimum in its fully relaxed state, with an energy gain of about an electron volt. However, the absence of Ta_2_O_2_(CH_4_)^+^ in the mass spectra suggests that this species reacts too fast to be stabilized by collisions with the He buffer gas–a similar effect was observed by us in the oxidation of small tantalum (oxide) cations^[^ ^40^ ^]^ or in the reaction of tantalum cations with CO_2_.^[^ ^41^ ^]^ Most significantly, the energetically hot intermediate can then react via a second C─H bond cleavage. This reaction has a certain barrier (0.30 eV with respect to the energy of the isolated reactants) if only the Ta‐atom inserts into the C─H bond. The process can however be strongly energetically facilitated when the hydride bound to Ta participates in the reaction by interacting with the hydrogen atom of the methyl group. In this pathway, H_2_ is directly formed on the cluster and the energy of this transition state is even below the entrance channel of the reaction (−0.13 eV relative to isolated Ta_2_O_2_^+^ and CH_4_). A similar but energetically less consequential observation was also made in the calculation of the atomic cation reacting with methane.^[^ ^12^ ^]^ From this structure, H_2_ can then desorb leaving Ta_2_O_2_(CH_2_)^+^ as the only charged reaction product, which is also observed in the experiment. The net energy balance of the first dehydrogenation of methane is approximately zero with the formed species being 0.02 eV more stable than the isolated starting species. The transition state of highest energy in the full potential energy surface is the insertion of the tantalum atom into the first C─H bond, which is in agreement with the high experimentally obtained KIE. Indeed, substituting hydrogen by deuterium in the calculations for this reaction step results in a similar value for the KIE of 5.3 (based on the respective Gibbs free energies, see Section 8 in the Supporting Information) as in the experiments (5.9 ± 1.7), further confirming this reaction step to be rate determining.

In addition, an interaction of the hydrogen atom of methane with the oxygen atoms of Ta_2_O_2_^+^ was also tested to evaluate the energetics of a HAT‐type reaction, as it occurs, for example for copper oxide catalysts^[^ ^42^ ^]^ (structures are given in Figures S10 and S11). However, it is found that this interaction is energetically unfavorable, which is in agreement with other tantalum clusters in low oxidation states^[^ ^17^ ^]^ but different to Ta_2_O_5_^+^.

**Figure 4:**
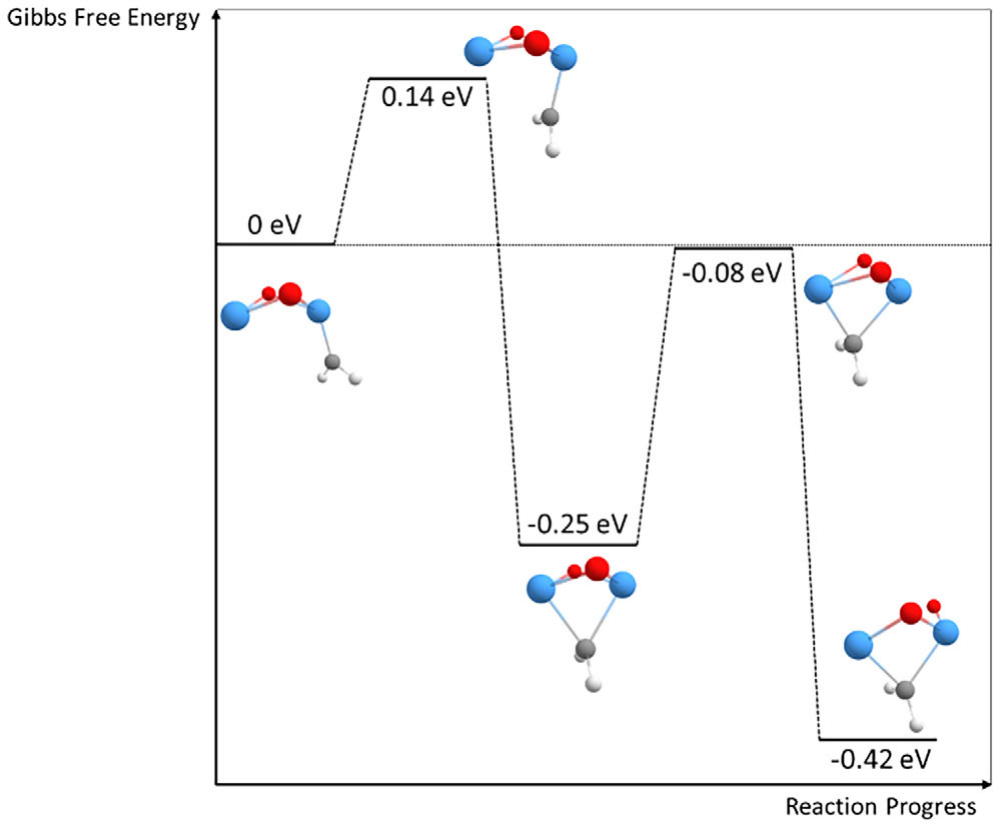
Energy scheme of a possible transformation of the final Ta_2_O_2_(CH_2_)^+^ cluster (Gibbs free energies calculated for *T* = 298.15 K and p = 1 atm). All energies are normalized to the unbridged cluster being 0 eV. By overcoming an energy barrier of 0.14 eV, a CH_2_ bridge between the two tantalum atoms is generated, leading to a more stable species. The cleavage of a Ta─O bond finally leads to the most energy‐efficient configuration with an energy of −0.42 eV. Tantalum atoms are shown in blue, oxygen atoms in red, carbon atoms in gray, and hydrogen atoms in white.

After the formation of Ta_2_O_2_(CH_2_)^+^ by this pathway, a more stable configuration can be created by bridging the two tantalum atoms with the CH_2_ group. This structure is obtained by a transformation (Figure 4), which has an energy barrier of only about 0.14 eV and yields a 0.25 eV more stable species. As the resulting conformer exhibits strained bonds, it can undergo a second transformation with a barrier of 0.08 eV below the initial structure to yield a more stable compound (−0.42 eV compared to the initial structure) in which one of the Ta─O bonds is cleaved. While tantalum compounds with more than one Ta‐atom generally favor motifs with carbenes bridging between two tantalum atoms,^[^ ^14^ ^]^ the reaction of Ta_2_O_2_^+^ with methane further shows that the formation of a second Ta─C bond is even energetically more favorable than the preservation of all Ta─O bonds.

**Figure 5:**
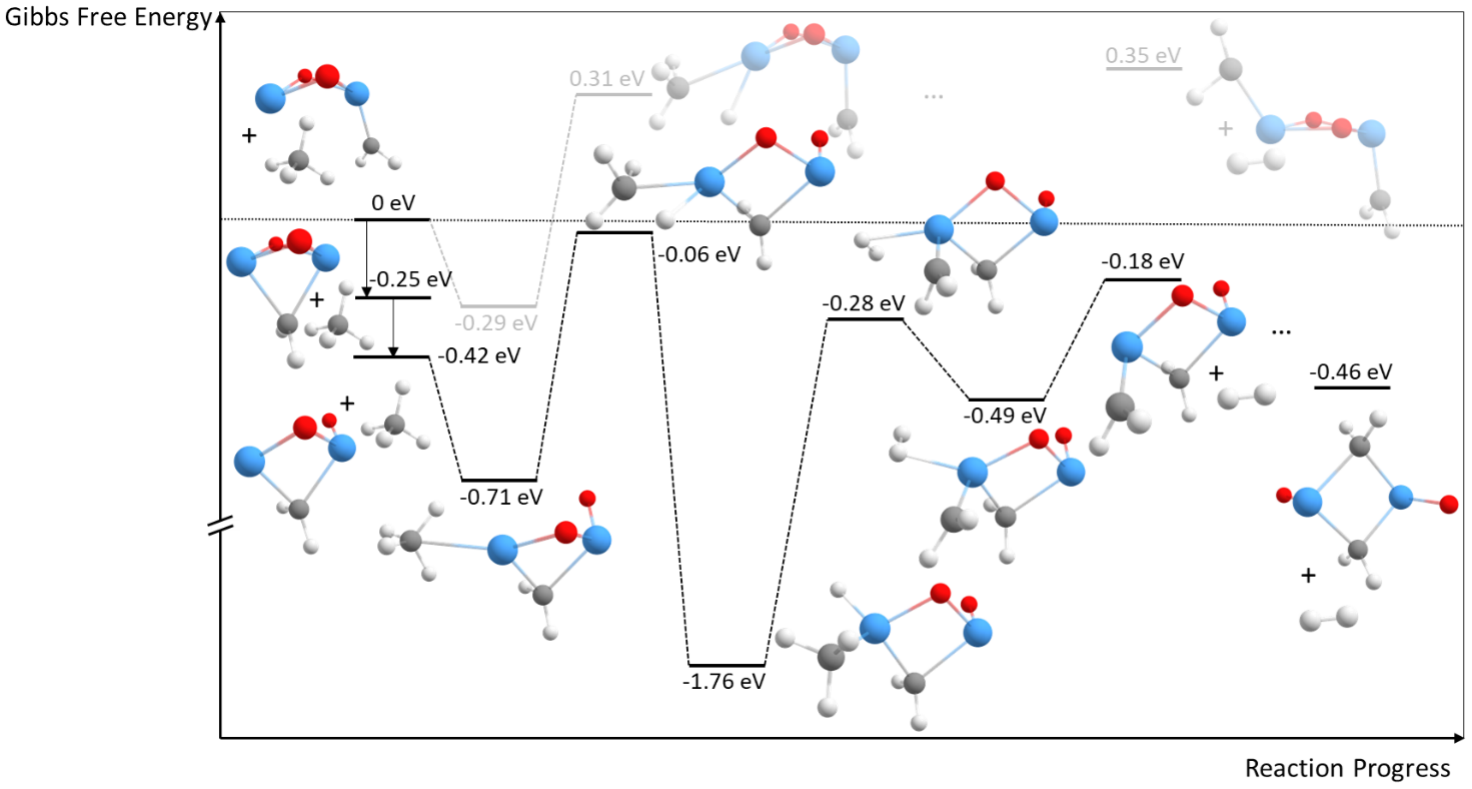
Energy scheme of the second dehydrogenation on Ta_2_O_2_(CH_2_)^+^ (Gibbs free energies calculated for *T* = 298.15 K and p = 1 atm). Energies are normalized so that the unbridged reaction product Ta_2_O_2_(CH_2_)^+^ from the first dehydrogenation reaction plus a free methane molecule equals zero. Tantalum atoms are shown in blue, oxygen atoms in red, carbon atoms in gray, and hydrogen atoms in white.

The existence of three possible product structures complicates the theoretical investigation of the second dehydrogenation reaction. Despite the higher complexity, possible pathways for the reaction can be obtained from DFT calculations (Figure 5). As a general behavior, structures with the carbon atom bridging between two Ta atoms are always energetically more favorable than unbridged compounds. While the latter may serve as reactive intermediates in the first step of the second methane dehydrogenation, they are becoming less and less energetically favorable as the reaction proceeds along its potential energy surface. Eventually, a dicarbene is yielded as the most stable compound, where both carbon and none of the oxygen atoms exhibit bonds to both tantalum atoms simultaneously. The transformation thereby occurs in an analogous manner as for the first carbene (compare Supporting Information Figure S9), exhibiting comparable energy barriers. The peculiar binding motifs make the resulting compound energetically even more favorable than the isolated reactants (i.e., Ta_2_O_2_(CH_2_)^+^ and CH_4_). The reaction can proceed without any transition state above the energy of the isolated reactants in the case of the unbridged structure following a similar pathway as in the reaction of the first CH_4_ molecule (see Figure S12 for comparison). However, a transformation into such a structure or a transition over a higher reaction barrier is required when the reaction starts from bridged reactants. This may give rise to the more sluggish reaction in comparison to the dehydrogenation of the first methane molecule, as the comparison of the rate coefficients of both reactions reveals (Table 1). Contrary to the atomic cation but in line with compounds with more Ta atoms.^[^ ^14^ ^]^ Our calculations show that C─C bond formations between two carbenes are not energetically favored. Typical candidate structures are between 0.54 and 0.87 eV higher in energy relative to isolated CH_4_ and the most stable configuration of Ta_2_O_2_(CH_2_)_2_^+^ (see Figure S8). Due to the absence of carbon chain formation, the dehydrogenation of further methane molecules is not enabled anymore on Ta_2_O_2_(CH_2_)_2_^+^ in which both Ta atoms are in a formal oxidation state of positive. Consequently, only the adsorption of intact CH_4_ is possible. Typically, this interaction leads to an energy gain in the range from 0.20 eV to 0.46 eV, as seen for Ta_2_O_2_^+^ (Figure 3) and Ta_2_O_2_(CH_2_)^+^ (Figure 5) as well as for all other adsorption steps in the Supporting Information (Figure S7).

### 3.2 Ta_2_O_5_^+^ + CH_4_ and the Origins of Reactivity

**Figure 6:**
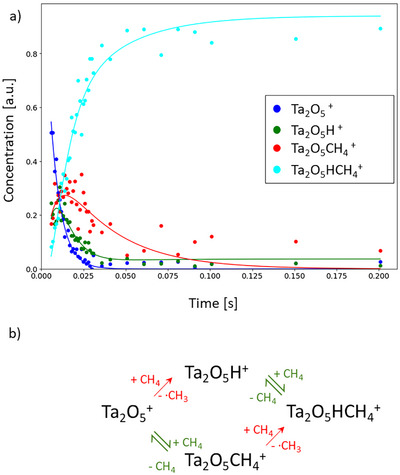
a) The fits of the kinetic simulations (lines) are shown in the respective color of the measured data points (dots) for each storage time. The fit is obtained by the reaction model shown in b).

In the mass spectra recorded in the interaction of methane with Ta_2_O_5_^+^, shown in Figure 1 only four species are identified, namely the bare metal oxide cluster Ta_2_O_5_^+^ (m/z = 442), Ta_2_O_5_H^+^ (m/z = 443), Ta_2_O_5_CH_4_^+^ (m/z = 458), and Ta_2_O_5_HCH_4_^+^ (m/z = 459). While the adsorption of an entire CH_4_ molecule onto the tantalum compound is again enabled, a second reaction scheme additionally occurs, which is the transfer of a single hydrogen atom onto the cluster and the release of a free methyl radical into the gas phase. The reaction via a HAT is completely different from the reactions of all other binary cationic tantalum (oxide) systems reported so far, which exclusively exhibit metal insertions into C─H bonds in the first step of methane activation.^[^ ^8^, ^9^, ^10^, ^11^, ^12^, ^13^, ^15^, ^16^, ^17^, ^18^ ^]^ Similar to the dehydrogenation in the case of Ta_2_O_2_^+^, it is assumed that this reaction takes place irreversibly due to the low degree of methane conversion, while adsorption of molecular methane is fitted as a reversible reaction step. The evaluation of different models reveals that the detected Ta_2_O_5_CH_4_^+^ is not an intermediate in the formation of Ta_2_O_5_H^+^, as this additional reaction channel does not lead to a significant improvement of the kinetic fits with the obtained rate coefficient for his pathway approaching zero (see Figure S4). Therefore, Ta_2_O_5_^+^ rather exhibits two separate reaction pathways with CH_4_, namely the HAT and the adsorption of methane, whereby one reaction is followed by the other one, leading to the same final product Ta_2_O_5_HCH_4_^+^. Applying this reaction sequence, a good agreement with the experimental data is obtained (Figure 6).

The bimolecular rate coefficients from the fits for the HAT reactions, together with the KIE from the reaction with CD_4_ are shown in Table 2 (for all values see Table S1). The HAT onto bare Ta_2_O_5_^+^ is only somewhat faster than the first dehydrogenation reaction on Ta_2_O_2_^+^. Its rate becomes slightly lower when CH_4_ is already adsorbed on the compound, but the decrease is not as significant as for the second dehydrogenation on Ta_2_O_2_^+^. Finally, the reaction yields the product Ta_2_O_5_HCH_4_^+^. The desorption of methane (i.e., the back reaction of intact CH_4_ adsorption) on this compound results in the occurrence of the second product Ta_2_O_5_H^+^, but only in minor amounts. As the concentration of CH_4_ is high relative to the concentration of the tantalum oxides, the equilibrium of this particular reaction is strongly shifted to the side of adsorbed methane with over 90% of the species carrying an intact CH_4_ molecule.

**Table 2:** Bimolecular rate coefficients given in 10^−1^° cm^3^s^−1^, and KIEs of the respective hydrogen atom transfer (HAT) reaction with the respective reactant (i.e., CH_4_ and CD_4_).

Reaction	$k_{+,CH4}^{\left(2\right)}$	$k_{+,CD4}^{\left(2\right)}$	KIE
Ta_2_O_5_^+^ → Ta_2_O_5_H^+^	5.5 ± 1.4	0.85 ± 0.20	5.9 ± 2.0
Ta_2_O_5_CH_4_^+^ → Ta_2_O_5_CH_4_H^+^	1.66 ± 0.50	0.401 ± 0.093	3.7 ± 1.4

**Figure 7:**
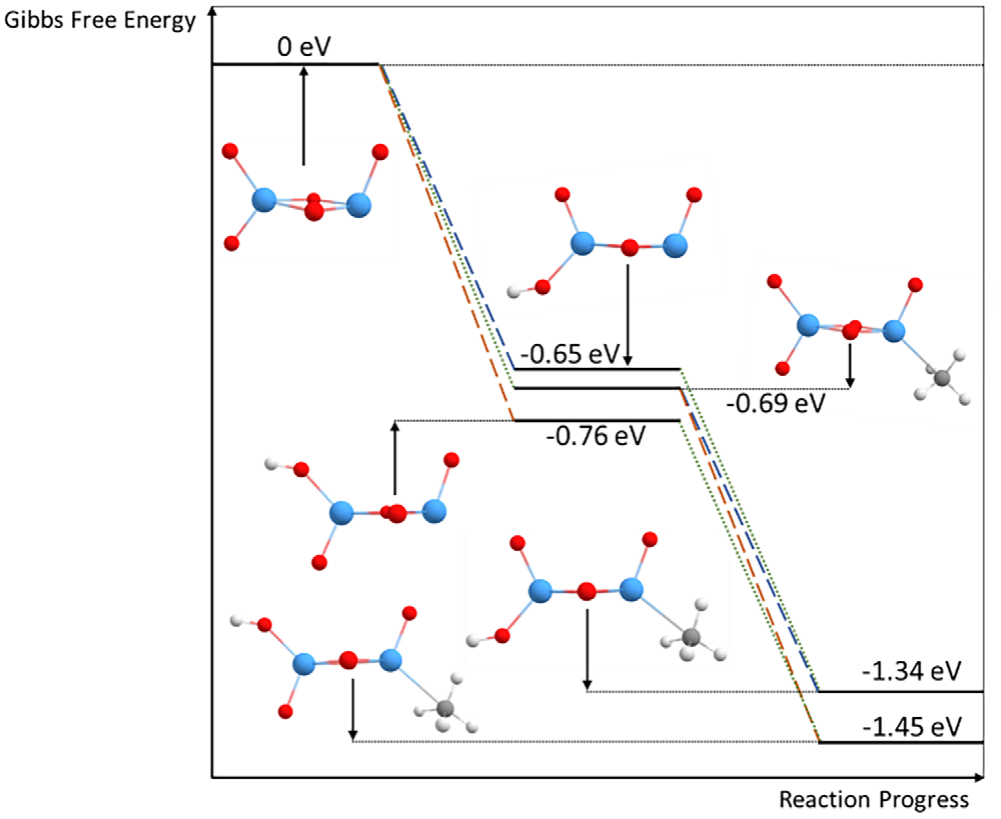
Energies obtained by DFT calculations for the reaction of Ta_2_O_5_^+^ with two methane molecules (Gibbs free energies calculated for *T* = 298.15 K and p = 1 atm). The first molecule can perform a hydrogen atom transfer (HAT) to the cluster or adsorb molecularly. The second molecule can then react following the respective other pathway. For the sake of clarity, free neutral molecules are not depicted in the energy diagram, while they are of course, included in the energy balance. The dotted lines show the adsorption of methane, while the dashed lines indicate the hydrogen atom transfer. The blue dashed line shows the addition of the hydrogen atom to the antioxygen, while following the orange line the hydrogen atom is added in syn‐position. The latter forms the more stable species. Tantalum atoms are shown in blue, oxygen atoms in red, carbon atoms in gray, and hydrogen atoms in white.

For deeper insights into the reaction mechanism, we again performed DFT calculations for this system. The structure of Ta_2_O_5_^+^ (Figure 7) is following the motif of oxygen addition described by Li et al. for smaller oxides of Ta_2_^+^ (i.e., Ta_2_O_x_^+^ with x = 0 – 4)^[^ ^18^ ^]^ and is similar to the anionic compound.^[^ ^43^ ^]^ For the bare metal oxide cluster, the doublet state proves to be the most stable multiplicity, which does not change by the adsorption of molecular methane. In HAT reactions, however, the overall parity already changes because the added hydrogen atom is a radical. Therefore, the singlet state is then identified as the most stable spin state for Ta_2_O_5_H^+^ and Ta_2_O_5_HCH_4_^+^ (see Supporting Information Table S4). The asymmetry of Ta_2_O_5_^+^ allows for different reaction centers to arise. Depending on the direction from which the methane molecule approaches the cluster, either a HAT to one of the oxygen atoms or molecular adsorption to a tantalum atom takes place. The molecular adsorption exclusively occurs at the tantalum atom bound to three oxygen atoms, while such a pathway is neither facilitated on the other tantalum atom nor at one of the other oxygen atoms according to our calculations. The adsorption leads to an energy gain of approximately 0.69 eV, significantly more than for the Ta_2_O_2_^+^ compounds. This may be attributed to a higher oxidation state of Ta in Ta_2_O_5_^+^, which corresponds to a higher positive partial charge on these atoms and favors the binding of CH_4_. For the hydrogen atom transfer, two different sites are available, where the hydrogen atom can either be added to a syn or antiposition regarding the oxygen atom bound only to the other tantalum atom. The reaction causes an energy change of −0.65 eV or −0.76 eV for the fully relaxed state (Figure 7). The exact value depends on the oxygen atom enabling the HAT, with the reaction being energetically preferred on the oxygen in syn‐position. Since the HAT is followed by molecular adsorption, similar to the molecular adsorption being followed by a HAT in the reaction scheme, Ta_2_O_5_HCH_4_^+^ is yielded as the final species, which is either 1.34 eV or 1.45 eV more stable than the isolated reactants. After the interaction with two methane molecules, both reaction sites on the tantalum compound are occupied and no further reactions are observed. Regardless of when the HAT takes place, the similar rate coefficients (see Table 2 and Supporting Information Table S1) of the reaction support the assumption that both reaction sites do not influence each other significantly.

**Figure 8:**
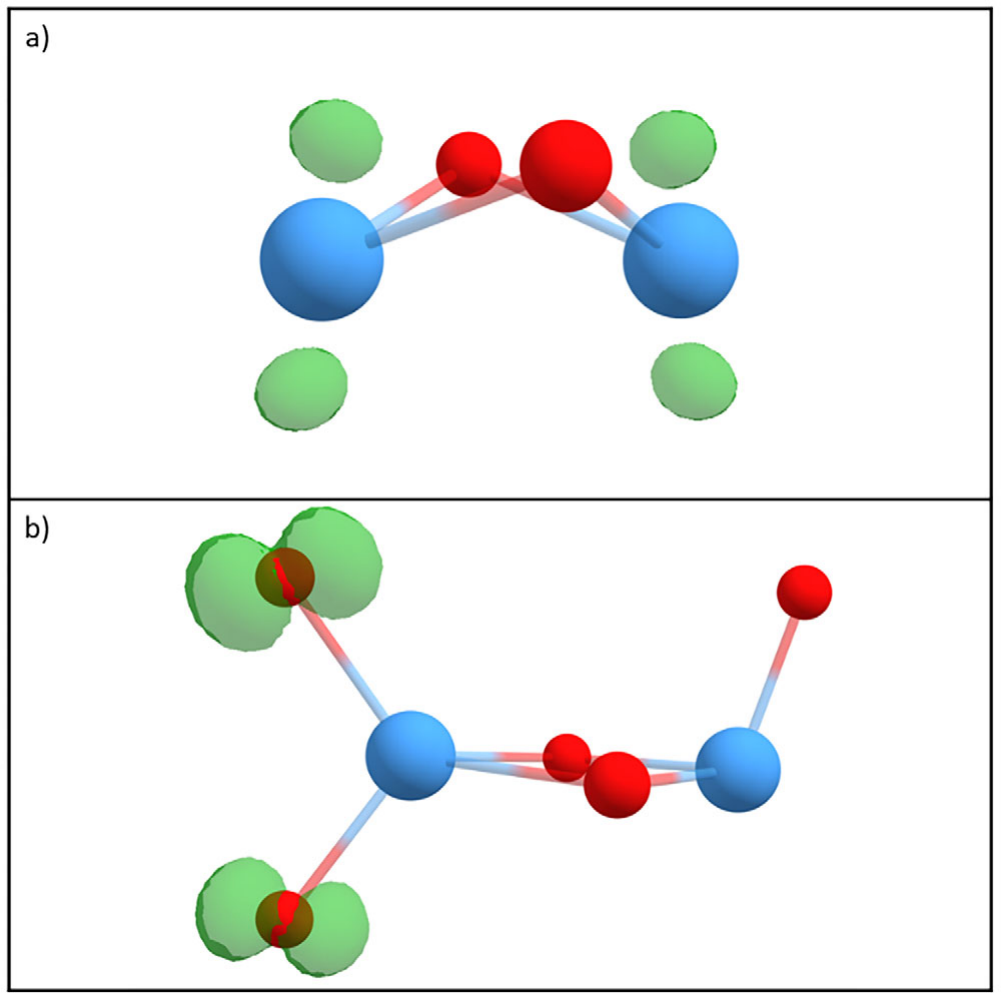
Spin densities (isosurface 0.03 e/Bohr^3^) on a) Ta_2_O_2_^+^ and b) Ta_2_O_5_^+^ shown in green. While for Ta_2_O_2_^+^ the spin density is located only on the tantalum atoms, a high spin density can be observed on two oxygen atoms on the Ta_2_O_5_^+^ cluster. Tantalum atoms are shown in blue, oxygen atoms in red.

The reason for metal oxides performing HAT reactions with methane has been assigned to the spin density on oxygen atoms in the reaction of different binary systems.^[^ ^19^, ^20^, ^23^, ^24^ ^]^ Consequently, spin densities may govern the reactivity of Ta_2_O_5_^+^ with methane too. Indeed, the comparison between bare Ta_2_O_2_^+^ and Ta_2_O_5_^+^ reveals significant differences in the electronic characteristics of both compounds. While the spin density is exclusively located on the tantalum atoms in Ta_2_O_2_^+^, the increased number of oxygen atoms in Ta_2_O_5_^+^ results in a significant spin density located on two of the oxygen atoms (Figure 8). Following the previously made assumption, it perfectly explains the observation of methane reacting via a HAT on Ta_2_O_5_^+^ but not on Ta_2_O_2_^+^, which instead enables the dehydrogenation of the molecule. The oxygen atoms in Ta_2_O_5_^+^ bearing the high spin density are the two bound end‐on to the same tantalum atom. In contrast to the remaining oxygen atom without a significant spin density, these oxygen atoms enable the HAT. The oxygen atom in syn‐position to the inert oxygen atom exhibits a higher spin density than the oxygen atom in antiposition, which explains the larger energy gain in the HAT reaction. In turn, the tantalum atom bound to the inert oxygen atom acts as binding site for intact methane adsorption so that the reactions are not sterically hindered.

**Figure 9:**
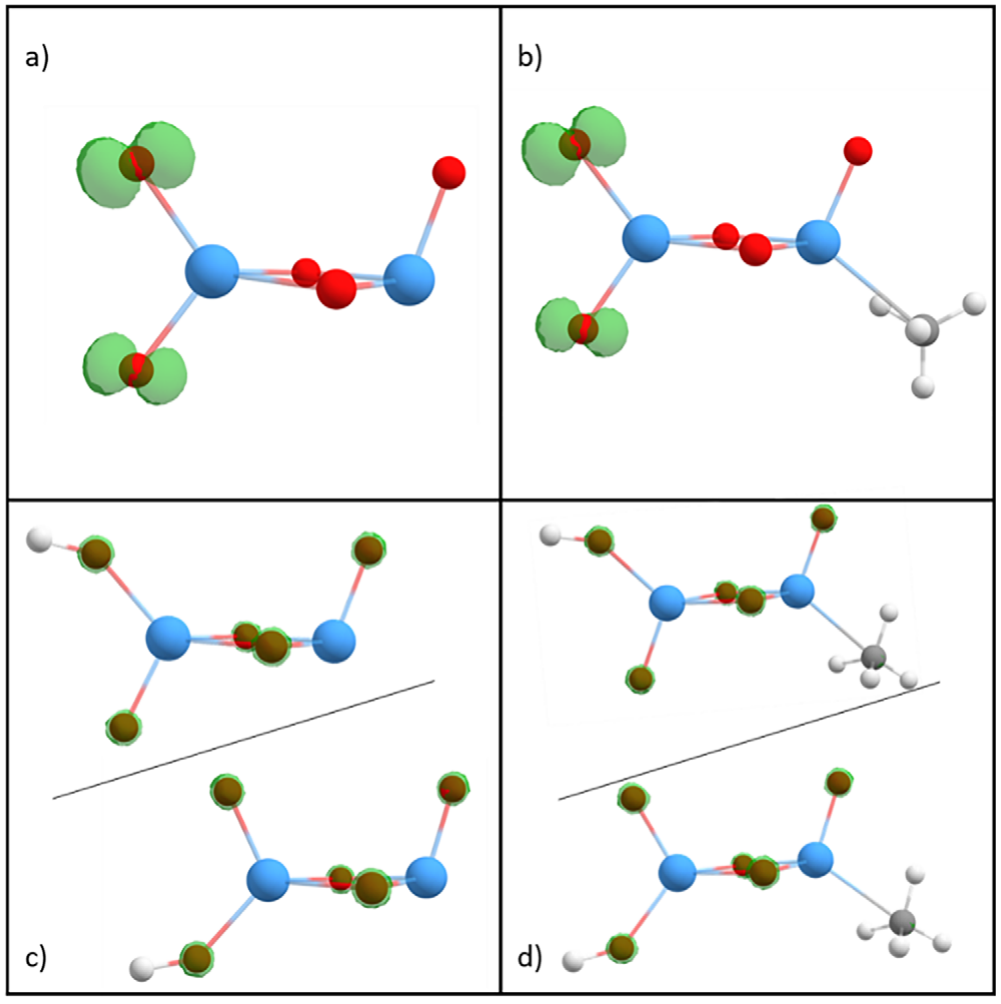
Spin densities (isosurface 0.03 e/Bohr^3^) on the Ta_2_O_5_^+^ cluster, shown in green, during the reaction with two methane molecules. a) shows the bare metal oxide cluster. In b), molecular adsorption of one methane molecule took place, which has no influence on the spin density. The hydrogen atom transfer in c) quenches any spin density including that on the other oxygen atom. This process also occurs when an intact methane molecule is adsorbed, so that the final product depicted in d) also exhibits no significant spin density. Tantalum atoms are shown in blue, oxygen atoms in red, carbon atoms in gray, and hydrogen atoms in white.

By each other. The spin density also remains very much unchanged upon intact methane adsorption (Figure 9), which results in the HAT being still enabled after this process. In contrast, the first HAT quenches the entire spin density, which was distributed over both oxygen atoms before so that a second HAT involving the other oxygen atom is not facilitated. Finally, with the adsorption site for methane occupied and no remaining spin density on any oxygen atom, Ta_2_O_5_HCH_4_^+^ does not exhibit any further reaction channels with methane anymore.

## 4 Conclusion

The study of cationic Ta_2_ compounds in the reaction with methane reveals the influence of their oxidation state on the reactivity. Different from previous studies,^[^ ^18^, ^19^ ^]^ we extend the oxygen contents to a level until the species deviate from their conventional “metallic” reaction behavior. Instead of a C─H bond insertion of a Ta atom, which is the common mechanism of the methane activation scheme of cationic tantalum compounds, usually leading to the dehydrogenation of the hydrocarbon,^[^ ^8^, ^9^, ^10^, ^11^, ^12^, ^13^, ^15^, ^16^, ^17^, ^18^ ^]^ Ta_2_O_5_^+^ enables the reaction via a HAT with the concomitant formation of a free methyl radical–a behavior typical for metal oxides for example, observed for V_4_O_10_^+^,^[^ ^19^, ^20^ ^]^ oligomeric (Al_2_O_3_)_x_^+ [^ ^23^ ^]^ or cationic ceria clusters of particular stoichiometries. Our calculations show that Ta_2_O_5_^+^ thereby comprises oxygen atoms with high spin densities, a typical characteristic for species enabling HAT reactions in the gas phase. Based on these findings, we speculate that the very stable compound (bulk) tantalum pentoxide^[^ ^44^ ^]^ may have the potential to enable the activation of methane, similar to MgO modified with lithium, which is already known in heterogeneous catalysis.^[^ ^45^, ^46^, ^47^, ^48^ ^]^ To make tantalum pentoxide active in the reaction with methane, oxygen radical centers (e.g., of dangling oxygen bonds) would likely have to be created on its surface, which may be done by particular treatments to achieve structural motifs with similar electronic characteristics as in Ta_2_O_5_^+^.

Instead, Ta_2_O_2_^+^ performs the dehydrogenation of methane, as it has been reported before.^[^ ^18^ ^]^ Our calculations demonstrate the relevance of Ta–CH_2_–Ta motifs in the reactions. On the one hand, the formation of such moieties seems to be detrimental to C─C bond formation in consecutive reactions with further methane molecules, but on the other hand, the stability of the resulting compounds may serve as a driving force for the reaction to proceed. As we have already speculated,^[^ ^7^ ^]^ this may lead to completely different product spectra in potential catalytic systems when single atoms or compounds with more Ta atoms are used and the results in here strengthen this assumption.

## Conflict of Interests

The authors declare no conflict of interest.

## Supporting information



Supporting Information

## Data Availability

The data supporting the findings of this study is available on the WWW under https://doi.org/10.5281/zenodo.14824549.
