# Supplementary material for: From C─H Bond Insertion to Hydrogen Atom Transfer: Tuning the Reaction Mechanisms of Methane Activation by the Oxidation of Ta2 +
Source: Chemistry. 2025 May 6;31(32):e202500545. doi: 10.1002/chem.202500545 (PMC12144900; doi:10.1002/chem.202500545)
Supplement: Supplementary file 1 — Supporting Information [file CHEM-31-e202500545-s001.pdf]

Table of Contents

Experimentally Obtained Reaction Rates for $Ta_2O_x^+$ (x = 0 – 6)………………………………………. 2

Test of Reaction Models……………………………………………………………………………… 4

Mass Spectra and Reactivity of $Ta_2O_2^+$/$Ta_2O_5^+$ with CD_4…………………………………………… 5

Adsorption Series of CH_4 on $Ta_2O_2^+$…………………………………………………………………… 7

CC-coupling on $Ta_2O_2^+$…………………………………………………………………………….. 7

CH_2 Bridging in $Ta_2O_2(CH_2)_2^+$…………………………………………………………………….. 8

Spin Multiplicities for Calculated Species………………………………………………………… 8

DFT Calculations with $Ta_2O_2^+$ + CD_4……………………………………………………………….. 10

HAT type reaction for $Ta_2O_2^+$……………………………………………………………………….. 13

Comparison of the First and Second Interaction of Methane with $Ta_2O_2^+$………………………….. 14

Adsorption Site on $Ta_2O_2(CH_2)^+$……………………………………………………………………...14

Theoretically Calculated xyz-Coordinates of All Species…………………………………………... 15

1. Experimentally Obtained Reaction Rates for $Ta_2O_x^+$ (x = 0 – 6)

All rate coefficients extracted for the kinetic fits are summarized in Table S1 together with available literature values from Li et al. [1], which show a very good agreement of the experimental results. While molecular methane adsorption reactions are usually fitted as reversible reactions, when the back reaction is strongly slower than the forward reaction, they are fitted as irreversible reaction steps.

Figure S1 is a graphical representation of the bimolecular reaction rate coefficients of $Ta_2O_x^+$ for the first interaction with methane and the reaction type.

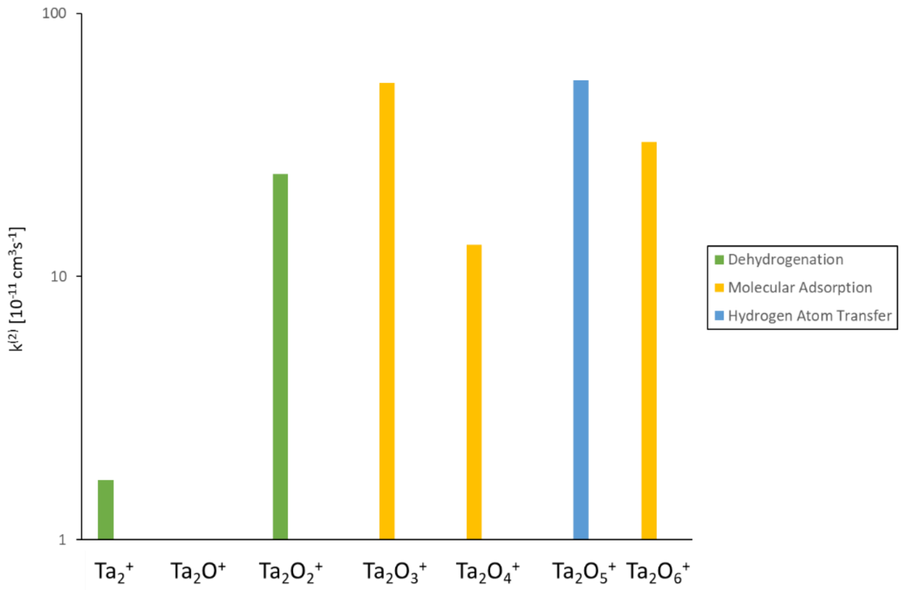

Figure S1. Bimolecular reaction rates of the interaction of the respective tantalum oxide cluster with the first methane molecule.

Table S1. Mono- and bimolecular rate coefficients for the reaction of $Ta_2O_x^+$ (with x = 0 – 6) with CH_4 and CD_4, respectively. k(1) is expressed in units of s^{-1}, while $k^{(2)}$ is given in units of 10^{-11} cm^3s^{-1}. Literature values are listed when available[1]. Kinetic isotope effects (KIE) are calculated for irreversibly fitted reactions.

Reaction	$k_+^{(1)}{}_{CH4}$	$k_-^{(1)}{}_{CH4}$	$k_+^{(1)}{}_{CD4}$	$k_-^{(1)}{}_{CD4}$	$k_+^{(2)}{}_{CH4}$	$k_+^{(2)}{}_{CH4,Lit}$	$k_-^{(2)}{}_{CH4}$	$k_+^{(2)}{}_{CD4}$	$k_-^{(2)}{}_{CD4}$	KIE
$Ta_2^+ \rightarrow Ta_2(CH_2)^+$	7.66 ± 0.08	-	12.2 ± 0.3	-	1.69 ± 0.34	1.1	-	0.175 ± 0.035	-	8.7 ± 1.9
$Ta_2(CH_2)^+ \rightarrow Ta_2(CH_2)_2^+$	45.9 ± 1.5	-	91.1 ± 6.8	-	10.1 ± 2.1	-	-	1.31 ± 0.28	-	6.9 ± 1.6
$Ta_2O^+ \rightarrow$ No Reaction	-	-	-	-	-	0.0032	-	-	-	-
$Ta_2O_2^+ \rightarrow Ta_2O_2(CH_2)^+$	79.2 ± 4.9	-	26.8 ± 0.5	-	24.5 ± 5.2	22	-	3.71 ± 0.75	-	5.9 ± 1.7
$Ta_2O_2(CH_2)^+ \rightarrow Ta_2O_2(CH_2)_2^+$	8.51 ± 0.68	-	3.35 ± 0.19	-	2.62 ± 0.57	-	-	4.65 ± 0.10	-	5.09 ± 2.23
$Ta_2O_2(CH_2)^+ \leftrightarrow Ta_2O_2(CH_2)(CH_4)^+$	19.8 ± 0.9	0.128 ± 0.110	16.2 ± 0.4	0.382 ± 0.153	6.11 ± 1.27	-	0.0395 ± 0.0349	2.25 ± 0.45	0.052 ± 0.026	-
$Ta_2O_2(CH_2)(CH_4)^+ \leftrightarrow Ta_2O_2(CH_2)(CH_4)_2^+$	5.64 ± 1.56	25.2 ± 7.5	1.35 ± 0.31	9.10 ± 2.73	1.74 ± 0.60	-	7.78 ± 2.81	1.87 ± 0.06	1.26 ± 0.46	-
$Ta_2O_2(CH_2)_2^+ \leftrightarrow Ta_2O_2(CH_2)_2(CH_4)^+$	32.9 ± 2.1	0.815 ± 0.461	21.6 ± 3.2	1.38 ± 0.91	10.2 ± 2.2	-	0.252 ± 0.151	2.99 ± 0.74	1.91 ± 0.13	-
$Ta_2O_2(CH_2)_2(CH_4)^+ \leftrightarrow Ta_2O_2(CH_2)_2(CH_4)_2^+$	3.78 ± 0.94	14.1 ± 4.0	-	-	1.16 ± 0.37	-	4.36 ± 1.51	-	-	-
$Ta_2O_3^+ \rightarrow Ta_2O_3(CH_4)^+$	89.1 ± 0.29	-	88.1 ± 0.4	-	54.3 ± 10.9	82	-	27.3 ± 5.5	-	1.8 ± 0.5
$Ta_2O_3(CH_4)^+ \rightarrow Ta_2O_3(CH_4)_2^+$	0.159 ± 0.010	-	0.219 ± 0.011	-	0.0971 ± 0.0521	-	-	0.0679 ± 0.0281	-	1.29 ± 0.87
$Ta_2O_4^+ \rightarrow Ta_2O_4(CH_4)^+$	107 ± 1	-	53.0 ± 0.4	-	13.2 ± 2.7	27	-	17.2 ± 3.5	-	0.7 ± 0.2
$Ta_2O_4(CH_4)^+ \rightarrow Ta_2O_4(CH_4)_2^+$	5.56 ± 0.35	-	2.12 ± 0.27	-	0.686 ± 0.144	-	-	0.691 ± 0.165	-	0.89 ± 0.28
$Ta_2O_5^+ \rightarrow Ta_2O_5H^+$	102 ± 15	-	21.3 ± 2.8	-	55.7 ± 13.8	-	-	8.47 ± 2.01	-	5.9 ± 2.0
$Ta_2O_5^+ \leftrightarrow Ta_2O_5CH_4^+$	55.3 ± 13.4	0.00 ± 9.22	52.2 ± 3.1	4.15 ± 1.99	30.1 ± 9.4	-	0.00 ± 3.71	20.8 ± 4.4	1.65 ± 0.86	1.3 ± 0.5
$Ta_2O_5H^+ \leftrightarrow Ta_2O_5HCH_4^+$	153 ± 15	6.03 ± 2.22	56.3 ± 8.4	7.77 ± 2.38	83.3 ± 18.6	-	2.41 ± 1.03	22.5 ± 5.6	3.10 ± 1.14	-
$Ta_2O_5CH_4^+ \rightarrow Ta_2O_5CH_4H^+$	30.5 ± 7.0	-	10.1 ± 1.2	-	16.6 ± 5.0	-	-	4.01 ± 0.93	-	3.7 ± 1.4
$Ta_2O_6^+ \rightarrow Ta_2O_4(CH_4)^+$	52.0 ± 0.6	-	91.2 ± 0.9	-	32.5 ± 6.5	-	-	29.7 ± 5.9	-	1.0 ± 0.3

2. Test of Reaction Models

Besides the final reaction mechanisms, different, chemically plausible, approaches have also been tested. The results for one alternative reaction model tested for the interaction of methane with $Ta_2O_2^+$ are shown in Fig. S2 together with the according monomolecular rate coefficients. Figure S3 shows the influence of fitting the dehydrogenation reaction reversibly, which results in the rate coefficients of the back reactions being zero, confirming the assumption of formally irreversible reaction steps.

Similarly, this was also done for the reaction of $Ta_2O_5^+$ with CH_4. Again, one of the tested models with the obtained rate coefficients is shown in Fig. S4.

a)

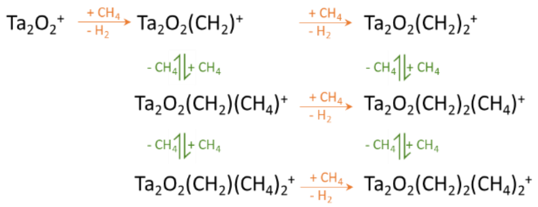

b)

Reaction	$k_+^{(1)}$	$k_-^{(1)}$	K
$Ta_2O_2^+ \rightarrow Ta_2O_2(CH_2)^+$	80.8 ± 5.0	-	-
$Ta_2O_2(CH_2)^+ \rightarrow Ta_2O_2(CH_2)_2^+$	8.86 ± 1.95	-	-
$Ta_2O_2(CH_2)^+ \leftrightarrow Ta_2O_2(CH_2)(CH_4)^+$	21.7 ± 2.0	0.506 ± 0.401	42.9
$Ta_2O_2(CH_2)_2^+ \leftrightarrow Ta_2O_2(CH_2)_2(CH_4)^+$	34.6 ± 3.2	1.14 ± 0.52	30.2
$Ta_2O_2(CH_2)(CH_4)^+ \rightarrow Ta_2O_2(CH_2)_2(CH_4)^+$	0.00 ± 2.38	-	-
$Ta_2O_2(CH_2)^+ \leftrightarrow Ta_2O_2(CH_2)(CH_4)_2^+$	7.13 ± 2.22	33.1 ± 17.3	0.22
$Ta_2O_2(CH_2)_2^+ \leftrightarrow Ta_2O_2(CH_2)_2(CH_4)_2^+$	5.37 ± 1.90	20.6 ± 8.4	0.3
$Ta_2O_2(CH_2)_2(CH_4)^+ \rightarrow Ta_2O_2(CH_2)_2(CH_4)_2^+$	0.0 ± 10.8	-	-

Figure S2. One alternatively tested reaction mechanism (a) and the respective monomolecular rate coefficients (b) given in s^{-1} for the interaction of $Ta_2O_2^+$ with CH_4.

a)

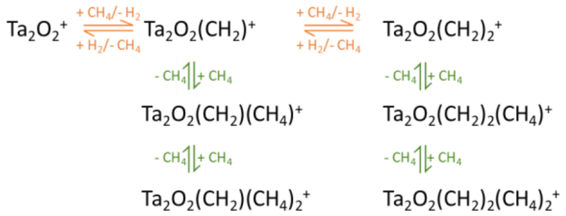

b)

Reaction	$k_+^{(1)}$	$k_-^{(1)}$	K
$Ta_2O_2^+ \leftrightarrow Ta_2O_2(CH_2)^+$	79.2 ± 4.9	0.0 ± 2.7	inf
$Ta_2O_2(CH_2)^+ \leftrightarrow Ta_2O_2(CH_2)_2^+$	8.51 ± 0.68	0.0 ± 7.1	Inf
$Ta_2O_2(CH_2)^+ \leftrightarrow Ta_2O_2(CH_2)(CH_4)^+$	19.80 ± 0.95	0.13 ± 0.11	154.3
$Ta_2O_2(CH_2)_2^+ \leftrightarrow Ta_2O_2(CH_2)_2(CH_4)^+$	32.9 ± 2.0	0.82 ± 0.46	40.4
$Ta_2O_2(CH_2)^+ \leftrightarrow Ta_2O_2(CH_2)(CH_4)_2^+$	5.6 ± 1.6	25.2 ± 7.5	0.223
$Ta_2O_2(CH_2)_2^+ \leftrightarrow Ta_2O_2(CH_2)_2(CH_4)_2^+$	3.78 ± 0.94	14.1 ± 4.0	0.267

Figure S3. Alternatively tested reaction mechanism including a reversible dehydrogenation reaction (a) and the respective monomolecular rate coefficients (b) given in s^{-1} for the interaction of $Ta_2O_2^+$ with CH_4.

a)

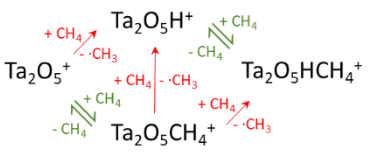

b)

Reaction	k_+(1)	k_-(1)	K
$Ta_2O_5^+ \longrightarrow Ta_2O_5H^+$	103 ± 15	-	-
$Ta_2O_5^+ \leftrightarrow Ta_2O_5CH_4^+$	53.7 ± 13.2	0.00 ± 9.22	Inf
$Ta_2O_5H^+ \leftrightarrow Ta_2O_5HCH_4^+$	153 ± 15	5.96 ± 2.22	26
$Ta_2O_5CH_4^+ \longrightarrow Ta_2O_5H^+$	0.0 ± 13.2	-	-
$Ta_2O_5CH_4^+ \longrightarrow Ta_2O_5HCH_4^+$	30.1 ± 7.0	-	-

Figure S4. One alternatively tested reaction mechanism (a) and the respective monomolecular rate coefficients (b) given in s^{-1} for the interaction of $Ta_2O_5^+$ with CH_4.

3. Mass Spectra and Reactivity of $Ta_2O_2^+$/$Ta_2O_5^+$ with CD_4

All reactions have been performed with CH_4 and CD_4 to ensure a correct assignment of the peaks. Figure S5 shows the mass spectra for $Ta_2O_2^+$ and $Ta_2O_5^+$, respectively, reacting with CD_4. All species experience the expected shift by the m/z of +1 for H -> D, +2 for CH_2 -> CD_2, and +4 for CH_4 -> CD_4, indicating a correct assignment of the compounds. Moreover, it shows that the reaction chamber is clean, since a) no residual hydrogen can be found on the clusters and b) the masses 16 corresponded to the addition of CH_4 and not an oxidation of the cluster.

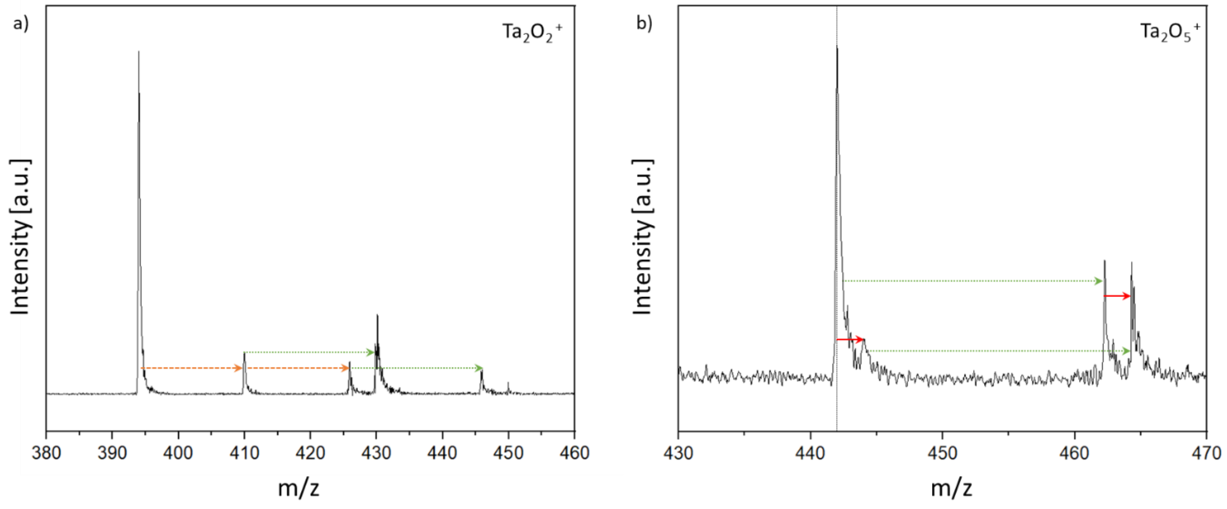

Figure S5. Mass spectra of a) $Ta_2O_2^+$ and b) $Ta_2O_5^+$. Orange (dashed) arrows indicate the dehydrogenation of methane, resulting in an addition of CD_2 (m/z = 16) to the cluster. Green (dotted) arrows show the adsorption of CH_4 (m/z = 20) and red (solid) arrows denote the addition of a single deuterium atom (m/z = 2). In b), the dotted black line serves as a reference of the center of the broad $Ta_2O_5^+$ peak. In order to show all present species, the mass spectra are an average over several storage times.

Using CD_4 further allows the investigation of the kinetic isotope effect. Figure S6 shows the kinetic fits of $Ta_2O_2^+$ and $Ta_2O_5^+$ with CD_4, respectively, using the reaction mechanisms stated in the manuscript. As the dehydrogenation rates decrease for the heavier isotope as a reactant, $Ta_2O_2(CD_2)_2(CD_4)_2^+$ is not formed, whereas $Ta_2O_2(CH_2)_2(CH_4)_2^+$ was found in small amounts of up to 10%.

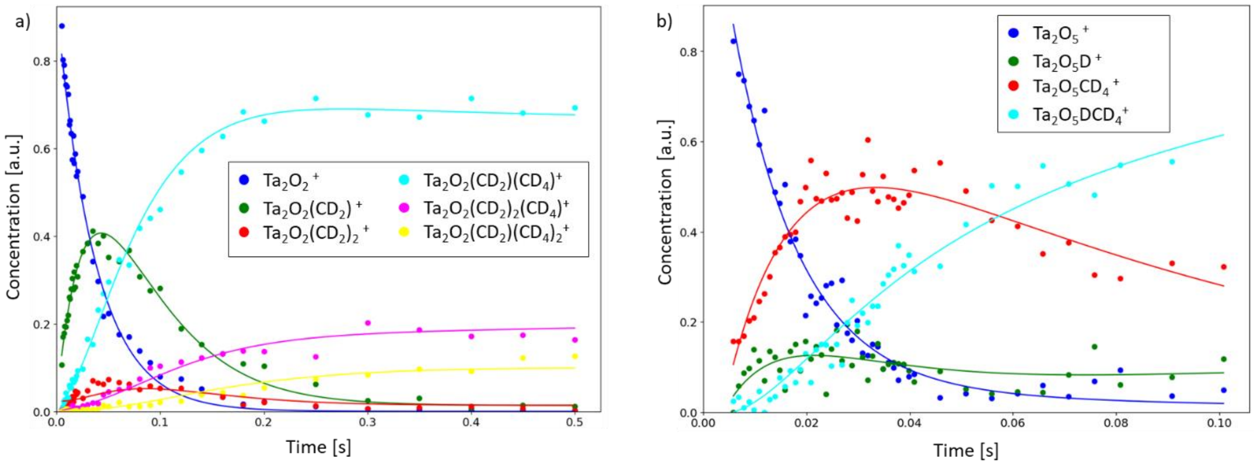

Figure S6. a) Kinetic fits (lines) of the reaction of $Ta_2O_2^+$ and b) $Ta_2O_5^+$ with CD_4. Measured data points are displayed as dots.

From the kinetic fits, the rate coefficients $k^{(1)}$ can be extracted. For a direct comparison, they have to be normalized to the methane particle density $[CH_4]$ and $[CD_4]$, respectively, to obtain the resulting bimolecular rate coefficients $k^{(2)}$:

$$k^{(1)} = k^{(2)} [CH_4] \quad (1)$$

The exact process of determining these rate coefficients, also including a potential unknown pressure gradient throughout the REIT, is described in detail by *Eckhard* et al. in their Supporting Information[2]. For the calculation of the kinetic isotope effect, the difference in collision probability of CH_4 and CD_4 have to be taken into account, so the kinetic isotope effect (KIE) is given by:

$$KIE = \frac{k_{CH4}^{(2)}}{k_{CD4}^{(2)}} \cdot 0.9 \quad (2)$$

The mono- and bimolecular reaction rates $k_+^{(1)}$ and $k_+^{(2)}$ of all forward reactions, as well as $k_-^{(1)}$ and $k_-^{(2)}$ for occurring backward reactions for both isotopes and the respective kinetic are listed in Table S1.

4. Adsorption Series of CH_4 on $Ta_2O_2^+$

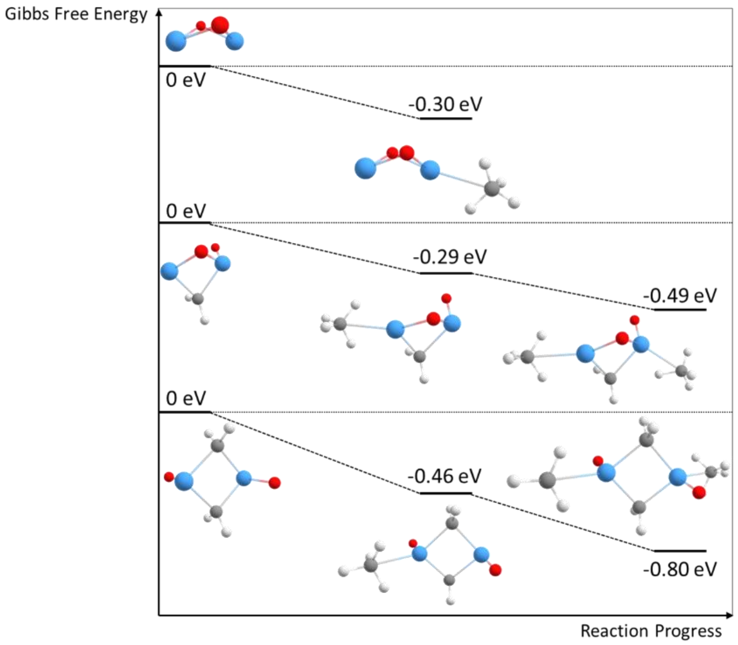

Figure S7. Energy gains by the adsorption of free methane molecules on $Ta_2O_2^+$, $Ta_2O_2(CH_2)^+$ and $Ta_2O_2(CH_2)_2^+$ (Gibbs free energies calculated for T = 298.15 K and p = 1 atm). For sake of clarity, the neutral molecules are not shown in the scheme, but taken into account in the energy calculations. Tantalum atoms are shown in blue, oxygen atoms in red, carbon atoms in gray and hydrogen atoms in white.

5. CC-coupling on $Ta_2O_2^+$

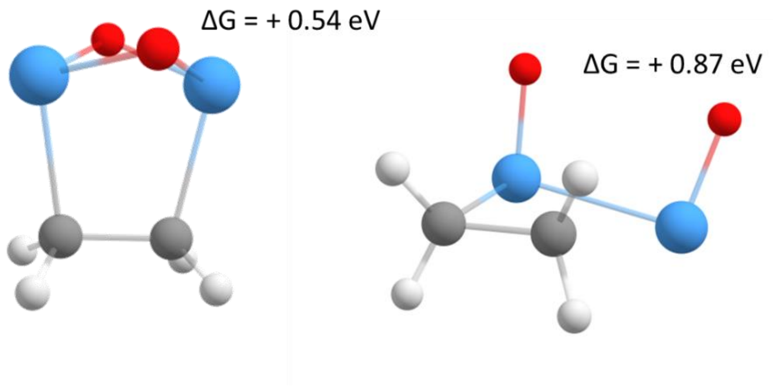

Figure S8. Optimized candidate structures with forced CC-coupling and their respective difference in Gibbs free energy compared to the most stable metal oxide cluster structure with two separate, bridging CH_2 groups (Gibbs free energies calculated for T = 298.15 K and p = 1 atm). Tantalum atoms are shown in blue, oxygen atoms in red, carbon atoms in gray and hydrogen atoms in white.

6. CH_2 Bridging in $Ta_2O_2(CH_2)_2^+$

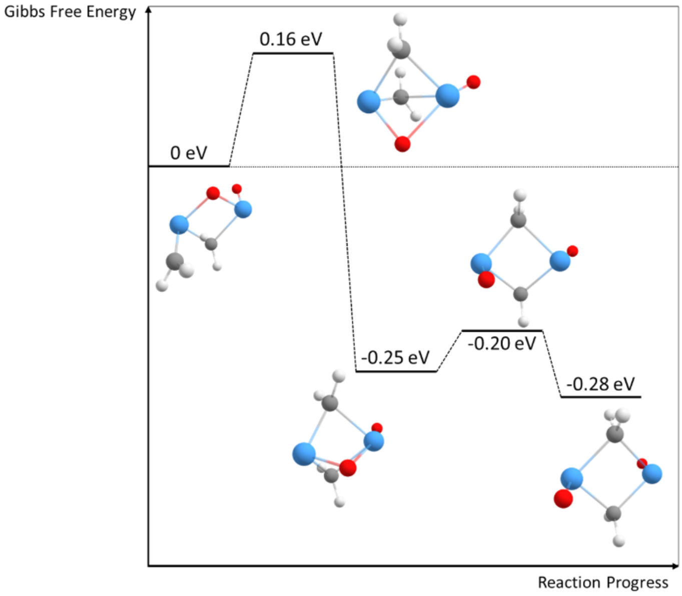

Figure S9. Transformation of the unbridged $Ta_2O_2(CH_2)_2^+$ into first the bridged form followed by the opening of the Ta-O-Ta bridge to minimize the Gibbs free energy (Gibbs free energies calculated for T = 298.15 K and p = 1 atm). Tantalum atoms are shown in blue, oxygen atoms in red, carbon atoms in gray and hydrogen atoms in white.

7. Spin Multiplicities for Calculated Species

For all species, structures, and energies for the three lowest spin multiplicities (namely doublet, quartet, and sextet for an odd number of electrons; singlet, triplet, and quintet for an even number) have been calculated to show that the lowest spin state is always the preferred one. Tables S2 – S4 summarize these calculations for $Ta_2O_2^+$, $Ta_2O_5^+$ and their reaction products, as well as reaction intermediates and transition states.

Table S2. Gibbs free energies for the different species in the two dehydrogenation reactions of $Ta_2O_2^+$ with methane for the three lowest spin densities. The doublet state proves to be the most stable one in all cases.

Species	Multiplicity	eV	Difference to Doub [eV]	Species	Multiplicity	eV	Difference to Doub [eV]
+ First Methane				+ Second Methane			
$Ta_2O_2^+$	Doublet	-7194.49	0.00	$Ta_2O_2(CH_2)^+$	Doublet	-8264.79	0.00
	Quartet	-7194.04	0.45		Quartet	-8264.15	0.64
	Sextet	-7193.66	0.84		Sextet	-8263.45	1.34
$Ta_2O_2(CH_4)^+$	Doublet	-8297.26	0.00	$Ta_2O_2(CH_2)(CH_4)^+$	Doublet	-9367.96	0.00
	Quartet	-8296.82	0.46		Quartet	-9367.60	0.36
	Sextet	-8296.46	0.80		Sextet	-9365.24	2.72
TS1	Doublet	-8298.11	0.00	TS1	Doublet	-9367.31	0.00
	Quartet	-8296.46	0.48		Quartet	-9366.37	0.94
	Sextet	-8295.77	1.17		Sextet	-9364.21	3.10
I1	Doublet	-8298.11	0.00	I1	Doublet	-9369.01	0.00
	Quartet	-8298.09	0.02		Quartet	-9366.62	2.38
	Sextet	-8295.19	2.92		Sextet	-9365.04	3.97
TS2	Doublet	-8297.09	0.00	TS2	Doublet	-9367.53	0.00
	Quartet	-8296.83	0.26		Quartet	-9366.46	1.26
	Sextet	-8294.77	2.31		Sextet	-9364.03	3.51
I2	Doublet	-8297.16	0.00	I2	Doublet	-9367.74	0.00
	Quartet	-8296.81	0.35		Quartet	-9366.46	1.28
	Sextet	-8295.56	1.60		Sextet	-9364.20	3.54
$Ta_2O_2(CH_2)^+$	Doublet	-8264.79	0.00	$Ta_2O_2(CH_2)_2^+$	Doublet	-9335.29	0.00
	Quartet	-8264.15	0.64		Quartet	-9334.41	0.89
	Sextet	-8263.45	1.34		Sextet	-9332.16	3.13

Table S3. Gibbs free energies for the different species in the transformation from the unbridged to the bridged CH_2 groups for the lowest three spin densities. The doublet state proves to be the most stable one in all cases.

Species	Multiplicity	eV	Difference to Doub [eV]	Species	Multiplicity	eV	Difference to Doub [eV]
$Ta_2O_2(CH_2)^+$ Transformation				$Ta_2O_2(CH_2)_2^+$ Transformation			
Unbridged	Doublet	-8264.79	0.00	Unbridged	Doublet	-9335.29	0.00
	Quartet	-8264.15	0.64		Quartet	-9334.41	0.89
	Sextet	-8263.45	1.34		Sextet	-9332.16	3.13
TS1	Doublet	-8264.65	0.00	TS1	Doublet	-9335.13	0.00
	Quartet	-8264.41	0.24		Quartet	-9333.46	1.67
	Sextet	-8263.58	1.07		Sextet	-9331.16	3.96
Bridged	Doublet	-8265.05	0.00	Bridged	Doublet	-9335.54	0.00
	Quartet	-8264.39	0.66		Quartet	-9333.86	1.68
	Sextet	-8263.49	1.55		Sextet	-9332.16	3.38
TS2	Doublet	-8264.79	0.00	TS2	Doublet	-9335.49	0.00
	Quartet	-8264.12	0.67		Quartet	-9333.36	2.13
	Sextet	-8263.09	1.70		Sextet	-9332.49	3.00
Open O	Doublet	-8265.21	0.00	Open O	Doublet	-9335.57	0.00
	Quartet	-8264.75	0.47		Quartet	-9333.86	1.74
	Sextet	-8262.43	2.79		Sextet	-9331.02	4.55

Table S4. Gibbs free energy for the different species in the reaction of $Ta_2O_5^+$ with up to two CH_4 molecules for the three lowest spin states. The doublet/singlet state proves to be the most stable one in all cases.

Species	Multiplicity	eV	Difference to Doub/Sing [eV]
+ First Methane			
$Ta_2O_5^+$	Doublet	-13345.08	0.00
	Quartet	-13342.28	2.81
	Sextet	-13338.85	6.22
$Ta_2O_5H^+$ (syn)	Singlet	-13363.80	0.00
	Triplet	-13360.99	2.81
	Quintet	-13357.96	5.84
$Ta_2O_5H^+$ (anti)	Singlet	-13363.70	0.00
	Triplet	-13361.01	2.69
	Quintet	-13357.96	5.74
$Ta_2O_5CH_4^+$	Doublet	-14448.26	0.00
	Quartet	-14444.91	3.34
	Sextet	-144441.19	7.06
+ Second Methane			
$Ta_2O_5HCH_4^+$ (syn)	Singlet	-14466.94	0.00
	Triplet	-14463.85	3.09
	Quintet	-14460.61	6.33
$Ta_2O_5HCH_4^+$ (anti)	Singlet	-14466.83	0.00
	Triplet	-14463.84	2.99
	Quintet	-14461.55	5.27

8. DFT Calculations with $Ta_2O_2^+$ + CD_4

To calculate the theoretically obtained KIE, the Boltzmann distribution has to be taken into account to calculate the property of a certain energy barrier to be overcome. Therefore, one can say:

$$KIE = f \cdot \frac{k_{CH4}^{(2)}}{k_{CD4}^{(2)}} = f' \cdot \frac{A_1 e^{-\frac{E_{A,1}}{kT}}}{A_2 e^{-\frac{E_{A,2}}{kT}}} = f \cdot e^{\frac{-E_{A,1} + E_{A,2}}{kT}}$$

Whereby the pre-factor f' includes e.g., the different collision cross sections of CH_4 and CD_4 and is known to be 0.9. Using

$$E_{A,2} = E_{A,1} + \Delta E_A$$

Equation (1) can be simplified to

$$KIE = 0.9 \cdot e^{\frac{\Delta E_A}{kT}}$$

The theoretically calculated energy barrier for overcoming the first transition state (normalized to $Ta_2O_2^+$ and the respective free methane molecule being 0 eV) equals 0.02 eV for CH_4 (compare Table S2 and the calculated Gibbs free energy of CH_4 being -1102.46 eV) and 0.07 eV for CD_4 (compare Table S5 with the calculated Gibbs free energy for CD_4 being -1102.86 eV), resulting in an energy difference of 0.05 eV between the first transition states of the reaction with CH_4 and CD_4. This leads to a KIE of 5.3. A list showing the difference in vibration frequency for H and D is given in S6. The respective energy contributions of the enthalpy, the zero point energy and the entropy can be seen in Table S7.

Table S5. Gibbs free energies of the species relevant for the first C-D bond activation in the reaction of $Ta_2O_2^+$ with CD_4. All energies are given for the doublet state.

Species	eV
$Ta_2O_2^+$	-7194.49
$Ta_2O_2(CD_4)^+$	-8297.60
TS1	-8297.23

Table S7. Energy contributions to the Gibbs free energy for the respective species in dependence of the used hydrogen isotope.

Species	Energy Type	Energy Contribution [eV]	
		X = H	X = D
$Ta_2O_2^+$	ΔH	-7193.46	
	ΔZPE	0.17	
	TΔS	1.03	
CX_4	ΔH	-1101.91	-1102.23
	ΔZPE	1.21	0.89
	TΔS	0.58	0.61
$Ta_2O_2(CX_4)^+$	ΔH	-8296.04	-8296.35
	ΔZPE	1.42	1.09
	TΔS	1.22	1.25
TS1	ΔH	-8295.75	-8296.02
	ΔZPE	1.26	0.98
	TΔS	1.18	1.22

Table S6. Vibrational frequencies for $Ta_2O_2(CX_4)^+$ and the first transition state in the reaction with the first methane molecule with X = H and D, respectively. Highlighted are vibrational modes which are strongly influenced by the exchange with the heavier isotope (i.e., C-X bond cleavage, methane deformation, and C-X stretching).

Species	Vibration Mode	Vibration Frequency [cm^{-1}]		Δ Frequency [cm^{-1}] (H vs. D)
		**X = H**	**X = D**	
**$Ta_2O_2(CX_4)^+$**	$Ta_2O_2^+$ deformation & vibrations related to the relative orientation of the molecules in space	36.04	32.96	3.08
		76.73	69.74	6.99
		164.84	119.92	44.92
		204.44	163.92	40.52
		270.73	234.53	36.20
		282.44	272.70	9.74
		293.79	292.63	1.16
		303.31	301.34	1.97
		407.08	403.59	3.49
		551.87	408.53	143.34
		746.26	745.89	0.37
		775.63	775.59	0.04
	**Methane Deformation**	**1238.84**	**935.87**	**302.97**
		**1342.84**	**1017.28**	**325.56**
		**1386.84**	**1031.08**	**355.76**
		**1523.25**	**1087.18**	**436.07**
		**1561.32**	**1110.20**	**451.12**
	**C-X Stretching**	**2705.35**	**1952.12**	**753.23**
		**2889.63**	**2130.96**	**758.67**
		**3066.62**	**2250.59**	**816.03**
		**3118.11**	**2317.30**	**800.81**

Species	Vibration Mode	Vibration Frequency [cm^{-1}]		Δ Frequency [cm^{-1}] (H vs. D)
		**X = H**	**X = D**	
**TS1**	**C-X Bond Cleavage**	**-1001.55**	**-730.92**	**-270.63**
	$Ta_2O_2^+$ deformation & vibrations related to the relative orientation of the molecules in space	61.78	55.17	6.61
		91.52	82.61	8.91
		192.28	138.83	53.45
		253.83	247.79	6.10
		282.49	249.14	33.35
		287.61	287.57	0.04
		395.04	345.93	49.11
		457.33	388.54	68.79
		478.39	476.13	2.26
	**Methane Deformation**	**717.46**	**568.23**	**149.23**
		**757.65**	**627.69**	**129.96**
	Rel. molecule orientation	793.25	740.92	52.33
		798.42	760.94	37.48
	**Methane Deformation**	**1205.16**	**920.53**	**284.63**
		**1357.67**	**989.17**	**368.50**
		**1449.48**	**1052.62**	**396.86**
	**C-X Stretching**	**1878.20**	**1336.54**	**541.66**
		**2846.39**	**2050.08**	**796.31**
		**2941.50**	**2184.07**	**793.43**
		**3117.10**	**2306.09**	**811.01**

9. HAT type reaction for $Ta_2O_2^+$

A relaxed surface scan has been performed for the doublet and the quartet $Ta_2O_2^+$ with CH_4 (see Fig. S10 and S11), whereby the distance between atom 2 and 5 has been varied between 1.5 and 1.0 Å in steps of 0.05 Å.

Bringing the hydrogen atom closer to the oxygen atom, resulting in the C-H bond cleavage, continuously leads to systems higher in energy, never forming a more stable intermediate. A HAT like this is thus not possible without overcoming reaction barriers >1.5 eV.

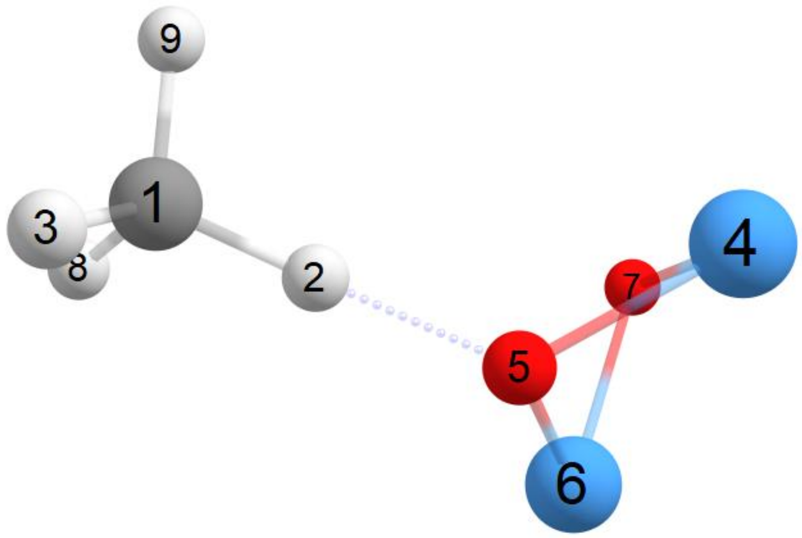

Figure S10. The oxygen atom of the doublet $Ta_2O_2^+$ interacting with the hydrogen atom of CH_4. Tantalum atoms are shown in blue, oxygen atoms in red, carbon atoms in gray and hydrogen atoms in white.

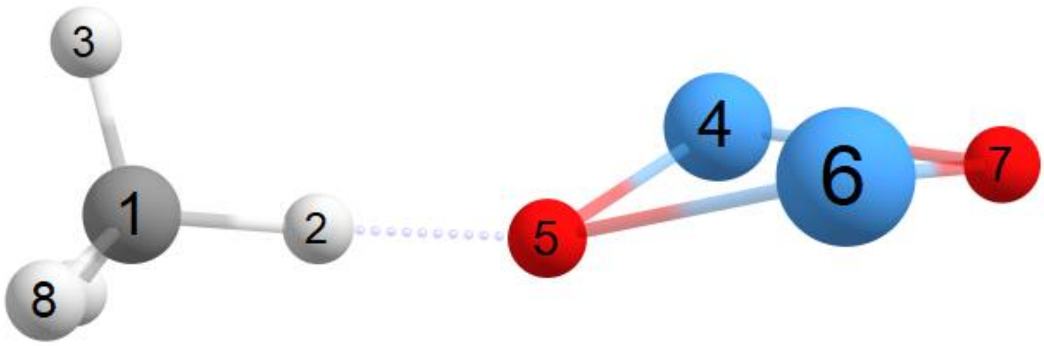

Figure S11. The oxygen atom of the quartet $Ta_2O_2^+$ interacting with the hydrogen atom of CH_4. Tantalum atoms are shown in blue, oxygen atoms in red, carbon atoms in gray and hydrogen atoms in white.

10. Comparison of the First and Second Interaction of Methane with $Ta_2O_2^+$

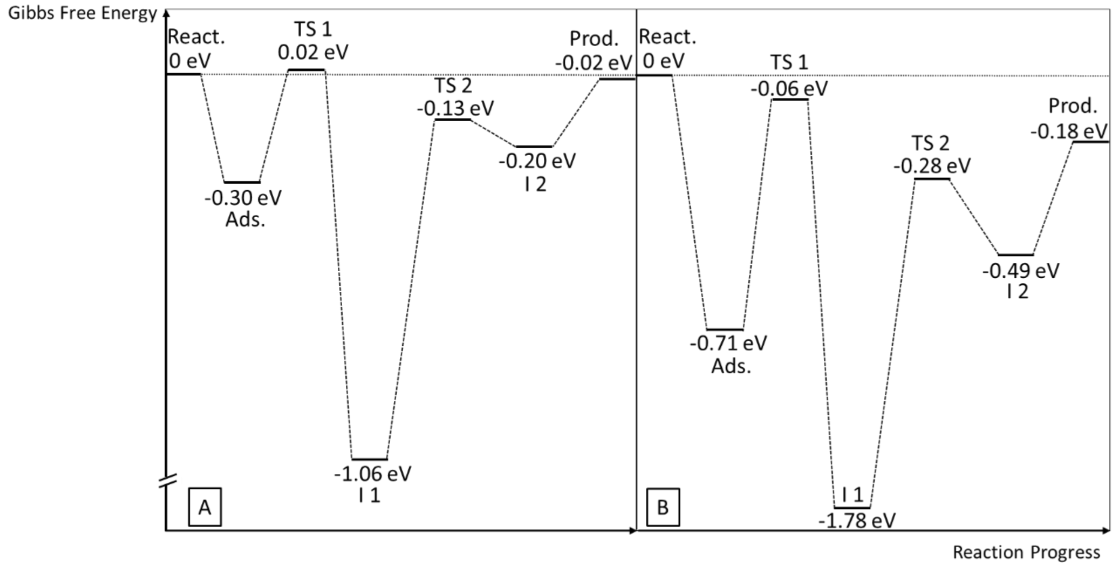

Figure S12. Comparison of the Gibbs free energies for A the first interaction with methane and B the interaction with the second methane molecule.

11. Adsorption Site on $Ta_2O_2(CH_2)^+$

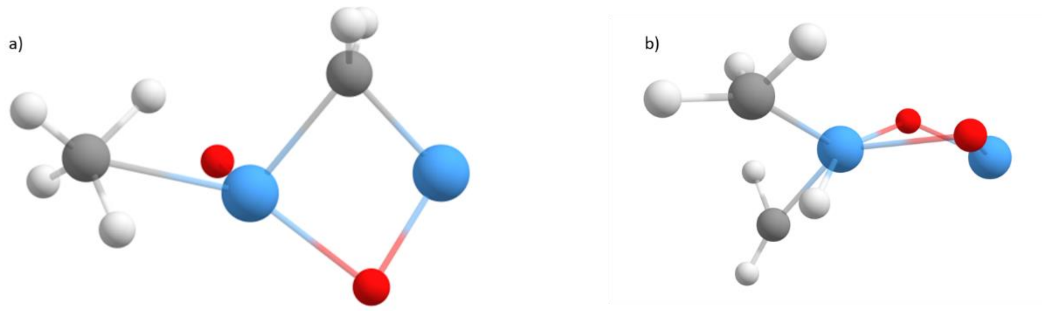

Figure S13. a) Structure of the molecular adsorption of methane on $Ta_2O_2(CH_2)^+$. b) Transition state of the C-H bond activation of the methane molecule. As this transition state of lowest energy is still too high in energy (0.74 eV above the entrance channel), a dehydrogenation of methane does not occur anymore. In addition, the intact methane molecule may prevent a restructuring of the compound.

12. Theoretically Calculated xyz-Coordinates of All Species

Species	Multiplicity	Element	x	y	z
$Ta_2O_2^+$	Doublet	Ta	-0.567597	-0.007282	-2.817323
		Ta	-0.812428	-0.057339	-0.433535
		O	-2.184480	-0.192016	-1.783074
		O	0.425024	-1.048573	-1.531590
	Quartet	Ta	-0.612411	-0.139722	-2.830540
		Ta	-0.847317	-0.176083	-0.436387
		O	-2.253851	-0.025762	-1.780869
		O	0.574098	-0.963645	-1.517725
	Sextet	Ta	-0.612881	-0.228994	-3.053356
		Ta	-0.883608	-0.200262	-0.224666
		O	-2.019952	-0.042388	-1.762540
		O	0.376961	-0.833568	-1.524960
$Ta_2O_2CH_4^+$	Doublet	Ta	-0.332886	-0.582559	-2.220318
		Ta	-0.652400	0.429846	-0.055423
		O	-1.967031	0.084037	-1.423670
		O	0.297681	-1.173532	-0.448627
		C	-0.867521	-0.358468	-4.689814
		H	-0.260784	-1.162144	-5.113610
		H	-0.235741	0.419502	-4.180428
		H	-1.697383	-0.758274	-4.066666
		H	-1.356096	0.199842	-5.492323
	Quartet	Ta	-0.838191	-0.450591	-2.328542
		Ta	-0.447786	0.203115	0.177462
		O	-2.028760	-0.012174	-0.759275
		O	0.410480	-1.062115	-0.864195
		C	-0.839701	-0.330719	-4.753347
		H	-0.220778	-1.230753	-4.571752
		H	-0.443668	0.582952	-4.233945
		H	-1.910564	-0.516877	-4.543830
		H	-0.753195	-0.084586	-5.813455
	Sextet	Ta	-0.684350	-0.225056	-2.290376
		Ta	-0.771824	-0.074040	0.564728
		O	-2.005947	-0.314304	-0.867116
		O	0.531364	-0.401958	-0.775285
		C	-0.822153	-0.408471	-4.838489
		H	-0.564092	-1.451883	-5.022533
		H	0.016740	0.167053	-4.373564
		H	-1.794939	-0.316294	-4.306789
		H	-0.976962	0.123206	-5.781455
$Ta_2O_2CH_4^+$ TS1	Doublet	Ta	-0.856460	-0.503644	-2.392650
		Ta	-0.304149	0.487495	-0.219474
		O	-2.032778	0.107728	-0.918464
		O	0.527842	-1.000526	-1.073905
		C	-0.933186	-0.514109	-4.613001
		H	-0.341849	-1.456092	-4.633126
		H	-0.419840	0.643632	-3.717163
		H	-2.022994	-0.726358	-4.616717

		H	-0.688747	0.060122	-5.506378
	Quartet	Ta	-0.931331	-0.438343	-2.486620
		Ta	-0.501273	0.219199	0.260680
		O	-1.769070	0.615401	-1.146041
		O	0.128038	-1.066504	-1.040491
		C	-0.835557	-0.485372	-4.643577
		H	-0.070881	-1.045746	-5.189323
		H	-0.084033	0.435251	-3.891266
		H	-1.611022	-1.292414	-4.227305
		H	-1.397031	0.156778	-5.326935
	Sextet	Ta	0.702970	0.615014	-1.560270
		Ta	-1.054492	-0.744969	0.237583
		O	0.407601	-1.164995	-0.981773
		O	-0.507367	1.068751	-0.186513
		C	-1.716592	-0.923169	-5.493226
		H	-1.427179	-1.133307	-4.469639
		H	0.234072	1.017305	-3.229348
		H	-1.689520	0.096501	-5.863059
		H	-2.021654	-1.732884	-6.144633
$Ta_2O_2CH_4^+$ I1	Doublet	Ta	-0.015463	0.185741	-2.360743
		Ta	-1.323718	-0.524246	-0.264861
		O	-1.660352	0.869403	-1.487771
		O	-0.595363	-1.594058	-1.564456
		C	1.936529	0.840595	-1.905751
		H	2.551020	0.055447	-1.444044
		H	2.331277	1.001958	-2.934281
		H	2.053393	1.767042	-1.332845
		H	-0.058232	-0.759470	-3.862660
	Quartet	Ta	0.075332	-0.042924	-2.289194
		Ta	-1.934716	-0.720798	-0.344581
		O	-1.352886	0.814106	-1.423746
		O	-0.365780	-1.518766	-1.217394
		C	1.985704	0.797400	-1.944893
		H	2.685208	0.204016	-1.344128
		H	2.361259	0.803968	-2.994161
		H	1.992229	1.833156	-1.582902
		H	-0.227259	-0.327746	-4.016414
	Sextet	Ta	0.064556	-0.178077	-2.379946
		Ta	-1.846839	-0.348980	-0.515388
		O	-1.399092	0.962194	-1.932244
		O	-0.281809	-1.458552	-1.006153
		C	2.204197	0.915050	-1.904130
		H	2.861932	0.053231	-2.040216
		H	2.196078	1.684538	-2.679329
		H	1.999816	1.232452	-0.881215
		H	-0.579748	-1.019444	-3.818791
$Ta_2O_2CH_4^+$ TS2	Doublet	Ta	-0.004583	0.389658	-2.158020
		Ta	-1.418108	-0.722820	-0.343069
		O	-1.776513	0.879338	-1.178748
		O	-0.632997	-1.499097	-1.837716
		C	1.866674	0.822377	-1.685519
		H	2.736338	0.309742	-1.275571
		H	1.566060	0.094908	-3.166157
		H	2.061126	1.902016	-1.823790
		H	0.821093	-0.333711	-3.688825
	Quartet	Ta	0.119652	-0.017392	-2.165570

		Ta	-1.862840	-0.666546	-0.215788
		O	-1.468476	0.774679	-1.435453
		O	-0.463400	-1.564861	-1.193447
		C	1.870870	0.781814	-1.673747
		H	2.763440	0.204154	-1.404680
		H	1.450048	0.410437	-3.499957
		H	2.055840	1.858601	-1.577852
		H	0.753959	0.061525	-3.990920
	Sextet	Ta	0.028314	0.027629	-1.973627
		Ta	-2.102992	-1.099821	-0.406642
		O	-1.641344	0.589081	-1.150023
		O	-0.586311	-1.712236	-1.396313
		C	2.199396	0.851788	-1.975039
		H	2.402970	0.902699	-0.900369
		H	1.369883	0.731267	-3.073928
		H	3.074686	1.121307	-2.569387
		H	0.474492	0.430698	-3.712084
$Ta_2O_2CH_4^+$ I2	Doublet	Ta	-0.105943	-0.814367	-2.384749
		Ta	-0.498704	0.697906	-0.397096
		O	-1.695336	-0.533804	-1.079506
		O	1.027936	-0.296953	-0.791214
		C	-0.035706	0.219079	-4.018916
		H	0.360245	1.059403	-4.582321
		H	1.577441	-1.852324	-2.870287
		H	-0.934264	-0.227490	-4.512041
		H	1.420498	-1.440850	-3.547229
	Quartet	Ta	0.274936	-0.677010	-2.230690
		Ta	-0.868709	0.628790	0.030866
		O	-1.297726	-0.839654	-1.141458
		O	0.842854	0.530791	-0.850928
		C	0.001554	0.115830	-4.022788
		H	0.632336	0.889524	-4.476589
		H	1.311679	-2.120065	-3.083481
		H	-0.868341	-0.104186	-4.654916
		H	1.087584	-1.613418	-3.753375
	Sextet	Ta	-0.145839	-0.645599	-2.399028
		Ta	-0.448129	0.207004	0.350835
		O	-1.565628	-0.151442	-1.125810
		O	0.952348	-0.386393	-0.726729
		C	-0.282315	0.073293	-4.390907
		H	0.491160	0.439051	-5.071639
		H	1.541295	-1.783644	-2.951301
		H	-1.272080	0.121796	-4.870756
		H	1.845355	-1.063465	-2.998023
$Ta_2O_2CH_2^+$	Doublet	Ta	-0.896366	-1.327541	-2.135505
		Ta	-0.572309	0.7440230	-0.845252
		O	-2.186712	-0.182420	-1.050055
		O	0.554588	-0.789785	-0.904415
		C	-0.701812	-0.834368	-4.004397
		H	0.049853	-0.414770	-4.671187
		H	-1.727566	-0.716233	-4.407316
	Quartet	Ta	-0.322960	0.096029	-2.539070
		Ta	-1.091666	-0.454676	0.177400
		O	-1.968316	-0.008901	-1.415852
		O	0.431630	-0.716914	-0.809170
		C	-0.733497	-0.883722	-4.088664

		H	-0.975749	-1.702926	-4.756721
		H	-0.819767	0.150016	-4.586052
	Sextet	Ta	-0.787733	-0.500541	-2.303014
		Ta	-0.606987	0.073005	0.524747
		O	-1.859414	0.294096	-0.899951
		O	0.460445	-0.728880	-0.840291
		C	-0.877233	-0.821924	-4.416104
		H	-0.086207	-1.282702	-5.021470
		H	-1.723196	-0.554148	-5.062043
Transformation TS1	Doublet	Ta	-0.897476	-1.522561	-1.999063
		Ta	-0.644333	0.713852	-1.233341
		O	-2.254492	-0.356111	-1.150467
		O	0.684720	-0.689870	-1.147013
		C	-0.808771	-0.758501	-3.815677
		H	0.126730	-0.555640	-4.345042
		H	-1.686699	-0.352263	-4.327524
	Quartet	Ta	-1.311449	-1.668865	-2.096976
		Ta	-1.415169	0.554788	-1.262374
		O	-2.918514	-0.576403	-1.714769
		O	-0.123839	-0.800045	-0.771291
		C	-0.678689	-0.855065	-3.778965
		H	0.367307	-0.603689	-3.977063
		H	-1.363132	-0.468760	-4.540596
	Sextet	Ta	-1.378653	-1.624349	-2.803268
		Ta	-0.547631	0.273952	-1.638020
		O	-2.404355	-0.131893	-2.001568
		O	0.172354	-1.521048	-1.575910
		C	-0.649856	-0.768930	-4.424643
		H	0.388674	-0.872267	-4.752236
		H	-1.206153	-0.0195058	-4.996193
CH_2 Bridged	Doublet	Ta	-0.435155	-0.637692	-2.139193
		Ta	-0.723816	0.228590	-0.010054
		O	-1.657735	0.822461	-1.638054
		O	0.948548	-0.505651	-0.746526
		C	-1.503550	-1.694439	-0.593600
		H	-1.038959	-2.587914	-0.169682
		H	-2.587111	-1.789525	-0.699170
	Quartet	Ta	-0.491665	-0.760589	-2.301729
		Ta	-0.786071	0.198254	0.215414
		O	-1.424814	0.732979	-1.518848
		O	0.715627	-0.348865	-0.857712
		C	-1.470444	-1.662960	-0.612844
		H	-0.982694	-2.560330	-0.216065
		H	-2.557715	-1.762659	-0.704495
	Sextet	Ta	0.807612	0.999757	-2.559223
		Ta	-0.819072	-0.553759	-0.754376
		O	-1.006327	0.950038	-1.952356
		O	1.033026	-0.405192	-1.279655
		C	-2.088238	-2.083227	0.028976
		H	-1.739788	-3.017909	0.485928
		H	-3.184990	-2.053878	0.034425
Transformation TS2	Doublet	Ta	-0.541271	-0.307828	-2.347141
		Ta	-2.443903	0.552266	-1.082697
		O	-2.283661	0.465511	-2.964031
		O	-1.043869	-1.521816	-1.155027
		C	-0.401259	1.208682	-0.848413

		H	-0.128196	2.223903	-1.158820
		H	0.057942	0.955016	0.115933
	Quartet	Ta	-0.546372	-0.605775	-2.517126
		Ta	-2.524556	0.142619	-1.298403
		O	-2.227162	0.275023	-3.160297
		O	-1.153015	-1.940153	-1.517662
		C	-0.495534	0.719447	-0.841777
		H	-0.185715	1.757652	-1.008306
		H	-0.112461	0.343117	0.115722
	Sextet	Ta	-0.084795	0.214706	-2.871060
		Ta	-2.327926	0.360523	-1.919382
		O	-1.671108	1.311046	-3.415283
		O	-0.761080	-1.418785	-2.723246
		C	-0.493536	0.646017	-0.818624
		H	-0.249747	1.644592	-0.437828
		H	-0.281295	-0.124781	-0.066296
Open O	Doublet	Ta	-0.632184	-0.409895	-2.247613
		Ta	-2.519129	0.930816	-1.084665
		O	-2.413328	0.479241	-2.84447
		O	-0.604916	-1.883510	-1.351774
		C	-0.446516	1.265494	-0.902875
		H	0.112263	2.170529	-1.175326
		H	-0.280407	1.023059	0.166531
	Quartet	Ta	-0.541194	-0.324542	-2.332192
		Ta	-2.603432	1.192159	-1.171757
		O	-2.394484	0.224000	-2.767236
		O	-0.642933	-1.735020	-1.361097
		C	-0.482036	1.193124	-0.821305
		H	-0.006642	2.134946	-1.152068
		H	-0.113495	0.891067	0.165459
	Sextet	Ta	-0.635641	-0.425145	-2.249705
		Ta	-2.649551	1.285381	-1.323122
		O	-2.476964	0.034474	-2.727645
		O	-0.655732	-2.030997	-1.625842
		C	-0.416245	1.367380	-0.727292
		H	0.076122	2.302665	-1.032857
		H	-0.026206	1.041977	0.246266
$Ta_2O_2(CH_2)(CH_4)^+$	Doublet	Ta	-0.041838	-0.549261	-2.242257
		Ta	-2.216231	0.706372	-1.587166
		O	-1.689896	0.155037	-3.261296
		O	-0.109148	-1.984638	-1.280575
		C	-0.245482	1.196240	-0.985402
		H	0.295341	2.132742	-1.175809
		H	-0.274114	0.998299	0.104461
		C	-4.149980	1.226711	0.047365
		H	-3.911984	0.164794	-0.218834
		H	-3.261786	1.826127	0.324691
		H	-4.739360	1.121243	0.961816
		H	-4.750622	1.724887	-0.718413
	Quartet	Ta	0.103306	-0.305860	-2.462666
		Ta	-2.400505	0.556866	-1.456005
		O	-1.704566	-0.019746	-3.136122
		O	0.217878	-1.844106	-1.694936
		C	-0.361719	1.019201	-0.855129
		H	-0.064599	2.067842	-1.034260
		H	-0.076151	0.693759	0.150669

		C	-4.142011	1.302669	0.072902
		H	-3.929136	0.230187	0.282012
		H	-3.267171	1.945315	0.299655
		H	-4.940247	1.595593	0.756347
		H	-4.530180	1.476833	-0.953887
	Sextet	Ta	0.133207	-0.265217	-2.481793
		Ta	-2.425457	0.434015	-1.519229
		O	-1.674041	-0.205018	-3.167067
		O	0.583297	-1.927985	-2.355383
		C	-0.390611	0.951129	-0.528951
		H	-0.210286	2.035084	-0.474011
		H	-0.002512	0.468462	0.377533
		C	-4.224658	1.423866	0.000894
		H	-3.609133	0.595776	0.423572
		H	-3.692353	2.071026	-0.742948
		H	-4.419459	2.093303	0.842417
		H	-5.163094	1.044112	-0.406452
$Ta_2O_2(CH_2)(CH_4)^+$ TS1	Doublet	Ta	-0.078302	-0.209642	-2.754978
		Ta	-2.204549	0.673104	-0.999751
		O	-2.024659	0.206253	-2.798838
		O	0.118168	-1.899397	-2.525848
		C	-0.159356	0.653138	-0.740778
		H	0.581257	1.294747	-0.254691
		H	-0.338539	-0.255956	-0.077178
		C	-3.934630	1.636237	-0.114309
		H	-3.819964	0.554294	0.329807
		H	-2.962362	2.350983	-0.841608
		H	-4.055146	2.307207	0.738522
		H	-4.835461	1.643429	-0.737501
	Quartet	Ta	-0.273039	-0.545991	-2.622295
		Ta	-1.976942	0.981350	-0.853350
		O	-2.091507	0.223204	-2.567816
		O	-0.304590	-2.158132	-2.028214
		C	0.157020	0.695948	-0.901269
		H	0.833561	1.562131	-0.943837
		H	0.332524	0.117754	0.029638
		C	-4.306309	1.258581	-0.143765
		H	-4.637916	0.225131	-0.257515
		H	-2.200085	2.736827	-0.740268
		H	-4.083995	1.608373	0.867433
		H	-4.706403	1.997893	-0.835690
	Sextet	Ta	0.069016	0.380620	-2.862687
		Ta	-2.439450	0.886227	-1.541192
		O	-1.488298	1.500048	-3.089545
		O	-0.184341	-1.159103	-3.582886
		C	-0.212487	0.021027	-0.687151
		H	0.194196	0.697146	0.077971
		H	-0.294290	-0.999780	-0.301761
		C	-3.640981	1.460615	0.532557
		H	-2.746182	1.133041	1.078934
		H	-4.177914	1.434813	-1.701889
		H	-3.821089	2.531265	0.481233
		H	-4.515863	0.817152	0.599469
$Ta_2O_2(CH_2)(CH_4)^+$ I1	Doublet	Ta	-1.286319	-0.025197	-2.220231
		Ta	0.311746	-0.850717	0.059520

		O	-2.366879	1.156802	-1.605722
		O	0.537768	0.227393	-1.439364
		C	-1.413622	-1.688180	-0.809148
		H	-1.392622	-2.722028	-1.188877
		H	-2.254005	-1.556974	-0.103576
		C	1.964481	-2.150622	0.246626
		H	2.816318	-1.947018	-0.411994
		H	1.671386	-3.205964	0.136573
		H	2.285325	-1.989093	1.295854
		H	0.014386	0.153657	1.488672
	Quartet	Ta	-1.435557	0.022761	-1.990928
		Ta	0.733126	-0.288433	-0.181793
		O	-2.529842	0.934224	-1.029287
		O	0.351444	0.711672	-1.798874
		C	-0.824467	-1.755188	-0.785982
		H	-0.621076	-2.566212	-1.505278
		H	-1.454136	-2.123236	0.028462
		C	1.380133	-2.375572	0.233244
		H	2.322362	-2.363851	-0.339463
		H	0.921816	-3.359650	0.203800
		H	1.522115	-2.060390	1.290890
		H	0.522045	0.625933	1.323542
	Sextet	Ta	-1.408520	-0.730473	-2.150206
		Ta	0.367153	-1.412216	0.063957
		O	-1.555377	0.972186	-2.358274
		O	0.458100	-1.283268	-1.815358
		C	-2.035986	-1.083887	-0.034357
		H	-2.644691	-1.998087	0.016262
		H	-2.669933	-0.200101	0.125263
		C	2.862492	-1.933640	0.148789
		H	2.933272	-1.798028	1.230635
		H	3.201418	-1.126168	-0.498171
		H	2.842367	-2.947901	-0.245730
		H	-1.462333	-1.056357	0.965524
$Ta_2O_2(CH_2)(CH_4)^+$ TS2	Doublet	Ta	-0.702009	-0.445782	-2.011930
		Ta	0.303568	-0.993752	0.744475
		O	-2.089991	0.554382	-2.124269
		O	0.331649	0.358486	-0.632606
		C	1.734459	-2.207660	0.483877
		H	1.043257	-3.027458	0.855298
		H	2.680238	-2.588287	0.111631
		C	-1.273287	-1.835086	-0.501014
		H	-1.075980	-2.865853	-0.832221
		H	-2.309963	-1.714140	-0.166063
		H	0.236116	-1.384551	2.816107
		H	-0.476077	-1.642125	2.597485
	Quartet	Ta	-0.993089	-0.244487	-1.853384
		Ta	-0.000613	-1.169883	0.404677
		O	-0.847154	0.600539	-0.190034
		O	0.658179	-0.944132	-1.709092
		C	1.899337	-2.015911	0.847461
		H	2.005854	-3.082413	1.097146
		H	2.873155	-1.517447	0.844234
		C	-1.656048	-1.989178	-0.856013
		H	-1.423238	-2.949884	-1.329601

		H	-2.656448	-1.989437	-0.404729
		H	-0.555819	-0.885347	2.322914
		H	-0.902137	-1.604247	2.167191
	Sextet	Ta	-1.191121	-0.061071	-1.497164
		Ta	0.227814	-1.310391	0.875225
		O	-0.961574	0.838869	-2.966516
		O	0.146075	0.298669	-0.123497
		C	2.046973	-2.235480	0.383827
		H	3.019550	-1.804238	0.657375
		H	2.157352	-3.160071	-0.192275
		C	-1.563340	-2.086218	-0.183710
		H	-1.499574	-2.572908	-1.183598
		H	-2.510826	-2.386476	0.277596
		H	-0.445113	-1.457963	2.836233
		H	-1.024238	-1.854549	2.457276
$Ta_2O_2(CH_2)(CH_4)^+$ I2	Doublet	Ta	-1.068051	-0.138764	-2.362334
		Ta	0.102081	-0.797622	0.220708
		O	-2.301244	1.026433	-2.091617
		O	0.505679	0.316739	-1.227727
		C	-1.475232	-1.697069	-0.878668
		H	-1.479398	-2.763135	-1.138696
		H	-2.436784	-1.426360	-0.400762
		C	1.586706	-2.058651	0.352434
		H	2.620285	-1.994619	0.006849
		H	1.339268	-3.057280	0.746241
		H	0.335100	-0.239746	2.157741
		H	0.948460	-0.785573	2.042978
	Quartet	Ta	-1.061159	-0.040225	-2.379710
		Ta	0.055621	-0.778026	0.182948
		O	-2.232836	1.165196	-2.014989
		O	0.569309	0.261966	-1.302705
		C	-1.482134	-1.652124	-0.981941
		H	-1.431253	-2.704360	-1.296864
		H	-2.438600	-1.459472	-0.458961
		C	1.698124	-2.098551	0.390529
		H	1.978184	-2.741712	-0.452549
		H	2.385285	-2.237765	1.231846
		H	0.067760	-0.361464	2.254932
		H	0.568570	-0.969110	2.254610
	Sextet	Ta	-1.408520	-0.730473	-2.150206
		Ta	0.367153	-1.412216	0.063957
		O	-1.555377	0.972186	-2.358274
		O	0.458100	-1.283268	-1.815358
		C	-2.035986	-1.083887	-0.034357
		H	-2.644691	-1.998087	0.016262
		H	-2.669933	-0.200101	0.125263
		C	2.862492	-1.933640	0.148789
		H	2.933272	-1.798028	1.230635
		H	3.201418	-1.126168	-0.498171
		H	2.842367	-2.947901	-0.245730
		H	-1.462333	-1.056357	0.965524
$Ta_2O_2(CH_2)_2^+$	Doublet	Ta	-0.921426	-0.120177	-2.140662
		Ta	0.232741	-0.893596	0.423481
		O	-2.167175	1.026044	-1.850743
		O	0.625595	0.288688	-0.951060
		C	-1.338109	-1.727264	-0.733883

		H	-1.342367	-2.775587	-1.049873
		H	-2.294237	-1.449846	-0.248869
		C	1.636730	-2.164191	0.163430
		H	2.532362	-2.258736	-0.446260
		H	1.365423	-3.094756	0.698513
	Quartet	Ta	-1.112588	0.154450	-2.109869
		Ta	0.120813	-1.082792	0.199184
		O	-2.274485	1.247481	-1.472584
		O	0.551725	0.262920	-1.030135
		C	-1.474158	-1.705146	-1.037279
		H	-1.422096	-2.664037	-1.573473
		H	-2.410576	-1.639405	-0.450744
		C	1.782766	-2.349328	0.392433
		H	2.150151	-3.068687	-0.344456
		H	2.417987	-2.324878	1.290998
	Sextet	Ta	-1.308044	0.261194	-2.011298
		Ta	0.394619	-1.548440	-0.427651
		O	-1.858105	1.429268	-0.878034
		O	0.566959	-0.171360	-1.725022
		C	-2.017362	-1.694156	-1.224658
		H	-2.184316	-2.563723	-1.876910
		H	-2.658573	-1.753070	-0.339350
		C	2.147171	-2.231048	0.576639
		H	3.170334	-2.218451	0.192311
		H	2.076853	-2.679636	1.578045
Transformation TS1	Doublet	Ta	-0.318247	-0.564474	-1.773802
		Ta	0.175692	-1.429030	0.897616
		O	-0.659944	-1.442038	-3.218879
		O	0.277464	-0.039008	1.925517
		C	-1.538262	-1.448454	-0.393014
		H	-2.162777	-1.739937	0.476543
		H	-2.408704	-1.434170	-1.071493
		C	1.454941	-1.284109	-0.756125
		H	2.303682	-1.386630	-1.444917
		H	1.205692	-2.401571	-0.777373
	Quartet	Ta	-0.572036	-0.112830	-1.518660
		Ta	0.167083	-1.186229	0.884021
		O	0.348716	-0.276842	-2.971395
		O	0.227770	0.558361	0.330658
		C	-1.662747	-1.672123	0.064849
		H	-1.917961	-2.719070	-0.134851
		H	-2.564320	-1.045021	-0.030754
		C	1.222128	-2.013868	-0.654880
		H	1.975710	-1.601295	-1.326875
		H	1.105193	-3.100508	-0.778042
	Sextet	Ta	-0.549067	-0.021240	-1.456940
		Ta	0.235098	-0.895596	1.036315
		O	0.101660	-0.682653	-2.924258
		O	0.454238	0.740612	0.190529
		C	-1.640233	-1.624469	-0.144075
		H	-1.720218	-2.524702	-0.769097
		H	-2.557628	-1.491509	0.442906
		C	1.119929	-1.999055	-0.576390
		H	2.191716	-1.861267	-0.761663
		H	0.694041	-2.809546	-1.173256
CH_2 Bridged	Doublet	Ta	-0.323645	-0.317909	-1.584411

		Ta	0.155320	-1.368977	0.938982
		O	-0.708722	-1.137743	-3.046479
		O	0.246758	0.376886	0.761211
		C	-1.601801	-1.447996	-0.202592
		H	-1.892195	-2.435824	-0.580840
		H	-2.489228	-0.865986	0.083117
		C	1.243378	-1.657794	-0.831072
		H	2.238930	-1.208727	-0.954815
		H	1.214514	-2.662696	-1.269257
	Quartet	Ta	-1.190822	0.421482	-1.390246
		Ta	0.252910	-1.147561	0.582203
		O	-0.355051	-0.013160	-2.831866
		O	0.067726	0.680099	0.098657
		C	-1.625599	-1.437366	-0.324558
		H	-1.815431	-2.426967	-0.752492
		H	-2.505073	-1.168158	0.307683
		C	1.585613	-2.432394	-0.415129
		H	2.248206	-2.143550	-1.236075
		H	1.667058	-3.501851	-0.174107
	Sextet	Ta	-1.310181	-0.421828	-0.955773
		Ta	0.104953	1.222721	0.988103
		O	-1.503946	0.190843	0.867798
		O	1.437229	0.199794	1.357159
		C	-1.938238	-2.421267	-1.286483
		H	-1.604767	-3.287041	-0.707583
		H	-2.596104	-2.693192	-2.123986
		C	0.233406	1.607370	-1.185287
		H	1.015878	1.212346	-1.841699
		H	-0.182210	2.537040	-1.600864
Transformation TS2	Doublet	Ta	-1.333085	-0.061316	-2.412416
		Ta	-0.621652	0.128442	0.391494
		O	-2.686551	0.766311	-1.728419
		O	0.862116	-0.521737	0.935246
		C	-1.230247	-1.518939	-0.887113
		H	-0.439011	-2.271833	-0.989261
		H	-2.166497	-1.983330	-0.547373
		C	0.111722	1.104805	-1.405431
		H	1.160888	0.855359	-1.606338
		H	-0.028673	2.194207	-1.372447
	Quartet	Ta	-1.550523	0.059042	-2.346339
		Ta	-1.185010	0.064114	0.474726
		O	-2.491781	0.833733	-1.001333
		O	0.477544	0.223602	0.958410
		C	-1.212891	-1.561212	-1.084759
		H	-0.227427	-2.017298	-1.253654
		H	-1.946333	-2.323989	-0.785271
		C	0.226563	0.965130	-1.585897
		H	1.152057	0.400752	-1.451471
		H	0.386812	2.048094	-1.546471
	Sextet	Ta	-0.386142	0.188671	-0.921398
		Ta	-0.777414	0.068013	2.332023
		O	0.058147	0.900828	0.764652
		O	-1.798694	-1.016678	1.453921
		C	-0.882687	-1.809131	-1.388850
		H	-1.124478	-2.609027	-0.686402
		H	-0.950610	-2.141716	-2.433794

		C	-0.203578	1.463439	-2.583153
		H	-0.395669	1.130064	-3.610262
		H	0.090136	2.517507	-2.548793
Open O	Doublet	Ta	-1.024243	-0.221390	-2.424528
		Ta	-0.258688	-0.035737	0.499996
		O	-2.542443	0.562486	-2.384456
		O	1.260592	-0.817827	0.472864
		C	-1.315781	-1.457592	-0.702727
		H	-0.805310	-2.428850	-0.741147
		H	-2.376259	-1.582206	-0.447764
		C	0.041119	1.195313	-1.226448
		H	1.104276	1.303524	-1.478380
		H	-0.454253	2.174251	-1.189469
	Quartet	Ta	-1.033199	-0.192274	-2.300405
		Ta	-0.242788	-0.068362	0.376220
		O	-2.566646	0.560468	-2.453012
		O	1.290658	-0.820925	0.529833
		C	-1.411160	-1.643950	-0.664188
		H	-0.904850	-2.616351	-0.713993
		H	-2.470332	-1.755589	-0.399475
		C	0.136579	1.382322	-1.261338
		H	1.196590	1.489987	-1.524593
		H	-0.365844	2.356646	-1.211107
	Sextet	Ta	-0.962944	-0.311708	-2.322764
		Ta	0.120970	-0.280313	0.274613
		O	-1.764562	0.990761	-3.167221
		O	1.527049	-1.210112	-0.079608
		C	-1.438194	-1.717949	-0.257628
		H	-1.222232	-2.743837	0.070418
		H	-2.511030	-1.597332	-0.463655
		C	-0.115173	1.451863	-1.201814
		H	0.804659	1.880471	-1.618333
		H	-0.809533	2.230125	-0.856066
$Ta_2O_2(CH_2)(CH_4)_2^+$	Doublet	Ta	-0.685580	-0.918871	-2.478967
		Ta	-2.599780	0.653956	-1.607878
		O	-2.330306	-0.041481	-3.306342
		O	-0.894882	-2.371503	-1.559189
		C	-0.595340	0.817933	-1.133761
		H	0.146919	1.617057	-1.044210
		H	-0.861509	0.455311	-0.099406
		C	-4.130812	1.590602	0.288078
		H	-4.122363	0.498433	0.041408
		H	-3.130647	2.066335	0.258183
		H	-4.439505	1.608035	1.336315
		H	-4.849856	2.137555	-0.324281
		C	1.789315	-0.859943	-3.157732
		H	1.788923	-1.952212	-3.195687
		H	1.351705	-0.408119	-4.060685
		H	2.824075	-0.508659	-3.104650
		H	1.376427	-0.446783	-2.194392
$Ta_2O_2(CH_2)_2(CH_4)^+$	Doublet	Ta	-0.998199	-0.186538	-2.447593
		Ta	-0.202897	-0.158606	0.480875
		O	-2.498961	0.631188	-2.595530
		O	1.267587	-1.016240	0.285512
		C	-1.421204	-1.421379	-0.723380
		H	-1.065911	-2.458232	-0.768973

		H	-2.492358	-1.392416	-0.479466
		C	0.046778	1.189499	-1.145989
		H	1.096767	1.389613	-1.392613
		H	-0.512084	2.130980	-1.050663
		C	-0.259877	-0.969420	-4.670996
		H	-0.405807	-1.884363	-4.061590
		H	-1.194905	-0.402188	-4.832364
		H	0.044914	-1.323912	-5.658200
		H	0.575319	-0.338781	-4.308590
$Ta_2O_2(CH_2)_2(CH_4)_2^+$	Doublet	Ta	-1.011573	-0.180146	-2.457307
		Ta	-0.301403	-0.086780	0.522765
		O	-2.538241	0.604131	-2.543967
		O	1.225726	-0.869658	0.605480
		C	-1.351078	-1.456349	-0.765460
		H	-0.847110	-2.431626	-0.793454
		H	-2.421619	-1.590928	-0.564191
		C	0.037420	1.188933	-1.171645
		H	1.108549	1.319324	-1.372827
		H	-0.462338	2.166167	-1.141466
		C	-0.296717	-0.957494	-4.702749
		H	-0.727241	-1.815676	-4.142016
		H	-1.017505	-0.132317	-4.852291
		H	-0.086733	-1.347223	-5.700917
		H	0.669026	-0.621153	-4.289516
		C	-0.970930	0.713686	2.780939
		H	-0.433882	-0.247105	2.898243
		H	-0.371209	1.491557	2.258641
		H	-1.101539	1.095783	3.795772
		H	-1.979048	0.599290	2.353734
Forced CC Coupling	Doublet	Ta	-0.826417	-0.905678	-1.818466
		Ta	-0.437551	0.684834	-0.118937
		O	-2.173435	0.117318	-0.824521
		O	0.578902	-0.957736	-0.454338
		C	-0.040716	0.760664	-2.992839
		H	0.851623	0.485476	-3.571260
		H	-0.804330	1.114035	-3.700337
		C	0.251238	1.790860	-1.873077
		H	-0.279366	2.742512	-2.011087
		H	1.323580	2.013896	-1.778741
Forced CC Alt 1	Doublet	Ta	-0.840912	-0.374406	-2.724213
		Ta	-1.119746	-0.793127	-0.051478
		O	0.770712	0.174199	-2.695639
		O	0.544670	-0.681857	0.284785
		C	-1.880355	1.471288	-2.628054
		H	-2.915419	1.415571	-2.978564
		H	-1.372075	2.369371	-2.969223
		C	-1.713523	1.139600	-1.148816
		H	-1.047829	1.834420	-0.629676
		H	-2.692921	1.070608	-0.641457
HAT to $Ta_2O_2^+$ TS1	Doublet	Ta	1.291695	-1.077396	0.001426
		Ta	2.472443	0.839148	-0.039166
		O	2.643862	-0.621556	-1.551991
		O	0.654780	0.619131	-0.708693
		C	5.566159	-1.197505	-1.048192
		H	3.538520	-1.053025	-1.526157
		H	5.677930	-1.189809	0.030768

		H	5.895050	-0.340883	-1.628242
		H	5.452620	-2.153330	-1.552863
	Quartet	Ta	1.224794	-1.460474	-0.982859
		Ta	1.609328	0.894930	-1.119187
		O	2.880849	-0.570524	-1.882182
		O	1.133637	-0.150396	0.433501
		C	5.553332	-1.030878	-0.766947
		H	3.813222	-0.699562	-1.524066
		H	5.271295	-1.038981	0.281295
		H	6.020615	-0.142115	-1.179923
		H	5.685989	-1.977223	-1.282744
$Ta_2O_2(CD_4)^+$	Doublet	Ta	-0.333182	-0.583164	-2.220221
		Ta	-0.651676	0.430082	-0.055425
		O	-1.966679	0.084935	-1.423442
		O	0.297031	-1.173980	-0.448287
		C	-0.867553	-0.358545	-4.689949
		D	-0.259919	-1.161489	-5.113830
		D	-0.236603	0.419930	-4.180221
		D	-1.697174	-0.759106	-4.067090
		D	-1.356407	0.199588	-5.492415
$Ta_2O_2(CD_4)^+$ TS1	Doublet	Ta	-0.856248	-0.500502	-2.394539
		Ta	-0.303461	0.484724	-0.217654
		O	-2.031968	0.115910	-0.921912
		O	0.521175	-1.007042	-1.071690
		C	-0.932959	-0.514317	-4.613720
		D	-0.332645	-1.450285	-4.634560
		D	-0.413037	0.645279	-3.717454
		D	-2.021167	-0.737406	-4.610041
		D	-0.701852	0.061888	-5.509307
$Ta_2O_5^+$	Doublet	Ta	-0.773114	0.898739	-1.570415
		Ta	0.384369	2.173048	-3.959367
		O	-1.188303	1.053581	-3.393135
		O	-1.779934	2.060385	-0.808372
		O	0.103329	3.878248	-4.542969
		O	0.780389	1.852071	-2.013377
		O	1.497053	1.377732	-5.087472
	Quartet	Ta	-0.843885	1.210704	-1.648668
		Ta	0.477791	2.163273	-4.083870
		O	-1.213310	1.205041	-3.471408
		O	-2.039027	1.638197	-0.198090
		O	0.272791	3.818028	-4.753084
		O	0.780100	2.011688	-2.073948
		O	1.589330	1.246874	-5.146041
	Sextet	Ta	-0.997263	1.515765	-1.600776
		Ta	0.512576	2.184844	-4.044357
		O	-1.197570	1.525877	-3.548116
		O	-2.050936	1.057732	-0.043842
		O	0.415035	3.887066	-4.899685
		O	0.815733	2.216908	-2.207054
		O	1.526215	0.905612	-5.031277
$Ta_2O_5CH_4^+$	Doublet	Ta	-0.909203	1.456426	-1.805903
		Ta	0.519552	2.513880	-4.147261
		O	-1.186368	1.583521	-3.672293
		O	-1.911468	2.646725	-1.066027
		O	0.445398	4.205463	-4.847926
		O	0.752315	2.281455	-2.173520

		O	1.624346	1.528593	-5.126283
		C	-1.113703	-0.667307	-0.563498
		H	-0.023932	-0.493835	-0.501886
		H	-1.446959	-0.969841	-1.573442
		H	-1.735763	0.123190	-0.089268
		H	-1.302936	-1.538110	0.072290
	Quartet	Ta	-0.811110	1.249251	-1.873293
		Ta	0.460625	2.480045	-4.227025
		O	-1.058577	1.417048	-3.908922
		O	-1.794342	2.494468	-1.221337
		O	0.107725	4.288674	-4.718849
		O	0.924231	2.203893	-2.423660
		O	1.679382	1.628139	-5.408456
		C	-1.182113	-0.587742	-0.356108
		H	-1.074310	-1.585499	0.076330
		H	-0.139325	-0.169167	-0.292505
		H	-1.893722	-0.006350	0.238738
		H	-1.566985	-0.798524	-1.391309
	Sextet	Ta	-0.509193	0.841451	-2.051013
		Ta	0.428205	2.582548	-4.343712
		O	-0.333584	0.715457	-4.035849
		O	-1.281534	2.416747	-2.037720
		O	-0.404310	4.078273	-3.579023
		O	1.224134	1.451445	-2.836501
		O	1.390202	2.751644	-5.980817
		C	-1.371032	-0.410507	-0.152643
		H	-1.552689	-1.194935	0.585838
		H	-0.276350	-0.194146	-0.043577
		H	-1.993572	0.459488	0.092883
		H	-1.668797	-0.883232	-1.124261
$Ta_2O_5H^+$ (syn)	Singlet	Ta	-0.798296	0.938287	-1.661251
		Ta	0.330232	2.430635	-3.930329
		O	-1.214036	1.228218	-3.463088
		O	-1.884444	1.948094	-0.793409
		O	0.053101	4.189886	-4.533121
		O	0.693327	2.018814	-1.993417
		O	1.442549	1.543020	-4.900771
		H	0.301666	4.833881	-5.213616
	Triplet	Ta	-0.879636	1.206590	-1.697215
		Ta	0.376532	2.477838	-4.018019
		O	-1.253749	1.361932	-3.507972
		O	-2.100165	1.383182	-0.210264
		O	0.175737	4.213015	-4.704121
		O	0.675037	2.165552	-2.023023
		O	1.469016	1.506240	-4.920219
		H	0.461328	4.816486	-5.408168
	Quintet	Ta	-1.096564	1.561579	-1.636120
		Ta	0.407275	2.414721	-4.015029
		O	-1.251607	1.615942	-3.618187
		O	-2.069915	0.965436	-0.071942
		O	0.455306	4.031884	-4.942576
		O	0.634747	2.396116	-2.145055
		O	1.595805	1.193829	-4.889090
		H	0.249052	4.951329	-5.171003
$Ta_2O_5H^+$ (anti)	Singlet	Ta	-1.238700	1.411961	-1.122260
		Ta	0.480839	0.862787	-3.448096

		O	-1.447997	0.765224	-2.867103
		O	-1.694746	3.065096	-1.206507
		O	0.731427	2.230489	-4.460351
		O	0.593667	1.400105	-1.508488
		O	1.369566	-0.684665	-4.049329
		H	1.946959	-1.005568	-4.759348
	Triplet	Ta	-1.167492	1.615303	-1.323216
		Ta	0.563013	0.821488	-3.546130
		O	-1.403129	0.871598	-3.008826
		O	-2.156691	3.056833	-0.515907
		O	0.869234	2.129493	-4.619693
		O	0.661453	1.517940	-1.632380
		O	1.400889	-0.778887	-4.053897
		H	1.973738	-1.188340	-4.721433
	Quintet	Ta	-1.144409	1.806789	-1.429007
		Ta	0.578552	1.002577	-3.672573
		O	-1.259649	1.048769	-3.263704
		O	-2.280920	2.366014	0.035655
		O	1.039718	2.270459	-5.031559
		O	0.774780	1.728467	-1.945713
		O	1.386231	-0.623630	-4.098330
		H	1.646713	-1.554015	-4.016252
$Ta_2O_5HCH_4^+$ (syn)	Singlet	Ta	-0.959678	1.296331	-1.704729
		Ta	0.689643	1.896080	-4.057371
		O	-1.127055	1.201086	-3.581375
		O	-1.928623	2.617366	-1.163265
		O	0.898023	3.452939	-5.101148
		O	0.767667	1.967558	-2.057647
		O	1.654369	0.566916	-4.589575
		H	1.361506	3.825270	-5.865595
		C	-1.335418	-0.668749	-0.254336
		H	-0.243476	-0.531131	-0.154318
		H	-1.630649	-1.062528	-1.244113
		H	-1.943660	0.192059	0.099298
		H	-1.592666	-1.459273	0.457375
	Triplet	Ta	-0.805793	1.071548	-1.787277
		Ta	0.828392	1.751098	-4.112293
		O	-0.831298	0.915500	-3.819529
		O	-1.705764	2.487360	-1.421619
		O	0.839471	3.364903	-5.056376
		O	2.056297	0.493394	-4.880060
		O	2.056297	0.493394	-4.880060
		H	0.650113	4.290591	-5.266282
		C	-1.491979	-0.538401	-0.153181
		H	-0.375752	-0.505820	-0.151134
		H	-1.933867	-0.877074	-1.120943
		H	-1.952775	0.388718	0.230340
		H	-1.729851	-1.319789	0.572097
	Quintet	Ta	-0.229853	1.692157	-1.277210
		Ta	0.960304	1.771523	-3.980066
		O	-0.143446	0.616566	-3.021700
		O	-0.753300	1.904772	0.576482
		O	0.382096	2.549131	-5.579702
		O	0.918795	2.828899	-2.420037
		O	2.675365	0.963239	-4.279172
		H	-0.214368	2.969733	-6.216056

		C	-1.793452	-0.388329	-0.594615
		H	-0.687030	-0.451507	-0.521472
		H	-2.195260	0.481462	-1.157233
		H	-2.209436	-0.371420	0.412622
		H	-2.100436	-1.272303	-1.158641
$Ta_2O_5HCH_4^+$ (anti)	Singlet	Ta	-1.308990	1.873137	-1.481181
		Ta	0.546948	0.897878	-3.542210
		O	-1.397530	0.833012	-3.053792
		O	-1.905367	3.444506	-1.866379
		O	0.804526	1.990446	-4.849622
		O	0.552013	1.882703	-1.794275
		O	1.516679	-0.717487	-3.697870
		H	2.139986	-1.156261	-4.296453
		C	-2.310250	1.175595	0.672006
		H	-2.667293	0.372958	0.001691
		H	-1.231801	1.098968	0.900026
		H	-2.818888	1.003541	1.625562
		H	-2.651145	2.195486	0.389883
	Triplet	Ta	-1.211304	1.762947	-1.348270
		Ta	0.537042	0.986089	-3.577588
		O	-1.301746	0.876299	-3.196293
		O	-1.599060	3.412254	-1.616939
		O	0.867558	2.398250	-4.805948
		O	0.780681	1.592348	-1.813950
		O	1.323383	-0.676320	-3.903537
		H	1.835724	-1.388418	-4.308035
		C	-2.363620	1.176640	0.675815
		H	-1.982813	0.149032	0.605669
		H	-1.556246	1.930999	0.863614
		H	-2.979043	1.239294	1.577197
		H	-3.081666	1.435065	-0.144349
	Quintet	Ta	-0.565640	1.207875	-1.949306
		Ta	1.168530	0.906451	-4.314339
		O	-0.540743	1.565464	-3.935138
		O	-1.617464	1.410665	-0.446658
		O	2.375274	2.324815	-4.800200
		O	1.254401	0.530087	-2.482617
		O	1.368642	-0.492436	-5.541565
		H	1.204047	-1.349670	-5.960481
		C	-3.254495	1.801386	1.950720
		H	-3.329806	0.745093	2.190273
		H	-2.530856	2.411651	2.482462
		H	-4.084374	2.287898	1.447413
		H	-2.178628	1.545200	0.366822

References

[1] Q. Li, Q.-Y. Liu, Y.-X. Zhao, S.-G. He, Conversion of Methane at Room Temperature Mediated by the Ta-Ta σ-Bond. *JACS Au* **2024**, 4, 1824.

[2] J. F. Eckhard, T. Masubuchi, M. Tschurl, R. N. Barnett, U. Landman, U. Heiz, Room-Temperature Methane Activation Mediated by Free Tantalum Cluster Cations: Size-by-Size Reactivity. *J. Phys. Chem. A* **2021**, 125, 5289.
